# All-optical permittivity-asymmetric *quasi*-bound states in the continuum

**DOI:** 10.1038/s41377-025-01843-9

**Published:** 2025-05-07

**Authors:** Rodrigo Berté, Thomas Possmayer, Andreas Tittl, Leonardo de S. Menezes, Stefan A. Maier

**Affiliations:** 1 Chair in Hybrid Nanosystems, Nanoinstitut München, Fakultät für Physik, Ludwig-Maximilians-Universität München, München, 80799 Germany; 2 Departamento de Física, Universidade Federal de Pernambuco, Recife, Pernambuco 50670-901 Brazil; 3 School of Physics and Astronomy, Monash University, Clayton, VIC 3800 Australia; 4 The Blackett Laboratory, Department of Physics, Imperial College London, London, SW7 2AZ UK

**Keywords:** Ultrafast photonics, Nanophotonics and plasmonics, Nonlinear optics

## Abstract

Resonances are usually associated with finite systems—the vibrations of clamped strings in a guitar or the optical modes in a cavity defined by mirrors. In optics, resonances may be induced in infinite continuous media via periodic modulations of their optical properties. Here we demonstrate that periodic modulations of the permittivity of a featureless thin film can also act as a symmetry-breaking mechanism, allowing the excitation of photonic *quasi*-bound states in the continuum (*q*BICs). By interfering two ultrashort laser pulses in the unbounded film, transient resonances can be tailored through different parameters of the pump beams. We show that the system offers resonances tunable in wavelength and quality-factor, and spectrally selective enhancement of third-harmonic generation. Due to a fast decay of the permittivity asymmetry, we observe ultrafast dynamics, enabling time-selective near-field enhancement with picosecond precision. Optically induced permittivity asymmetries may be exploited in on-demand weak to ultrastrong light-matter interaction regimes and light manipulation at dynamically chosen wavelengths in lithography-free metasurfaces.

## Abstract (web summary)

Tailored ultrafast resonances (in amplitude, Q-factor, wavelength) in an unpatterned thin Si film driven by permittivity asymmetries. Spectrally selective third-harmonic generation (THG) enhancement is shown accordingly.

## Graphical abstract

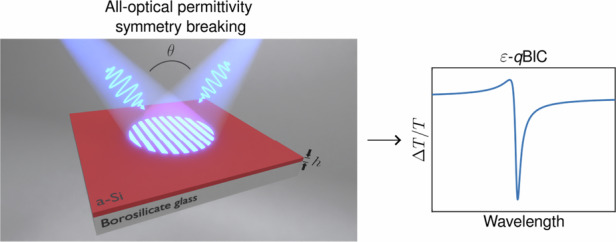

## Introduction

While individual atoms display spectral lines^1^, electronic and phononic interactions in extended matter generate broad optical responses^2^. Bulk excitonic resonances are a prominent exception, but their wavelength is predefined near the band edges of the given material^3^, severely limiting the optical bandwidth available for applications. A new paradigm in optical resonances appeared with the ability to pattern materials below or in the same size scale as the wavelength of light^4^, into flat (metasurfaces^5^) and into three-dimensional (metamaterials^6^) structures, in which optical functionalities are not limited by the weakly-dispersive macroscopic permittivity of its constituents. However, their optical response is intimately linked to the dimensions of the comprising resonators established during fabrication.

Optical modes in infinite featureless media have been shown to arise from periodic modulations of the permittivity of thin films in the seminal theoretical works on guided-mode resonances (GMRs)^7–9^, in which narrower linewidths were observed for vanishing permittivity modulations (Δ*ε* → 0). Periodic modulations, recently explored in time-varying metamaterials^10^, have also been extensively investigated using laser beam interference in dynamic gratings induced in featureless films^11^. Such induced gratings are commonly measured via diffraction effects that are almost invariant for small changes in the wavelength of the probing beam.

Arising in the context of electronic wavefunction localization^12^, BICs are a set of phenomena demonstrated in elastic^13^, acoustic^14^, hydrodynamic^15^, and optical^16^ systems, where particular oscillatory excitations remain spatially localized in spite of co-existing in the continuum of possible propagating modes. True BICs can only exist in systems devoid of radiative losses and that are, generally, spatially infinite in at least one dimension^17^. Among the different types of BICs^17^ are those whose symmetry is incompatible with that of propagating modes^18^, thus termed symmetry-protected BICs. Since true BICs cannot be observed due to the strict requirement of an infinite system^17^, experimental studies have focused on measuring localized excitations corresponding to BIC conditions via electron energy loss spectroscopy and near-field coupling in finite arrays^19,20^. In finite photonic systems, their far-field observable counterparts, called *quasi*-BICs (*q*BICs)^21^, are usually probed via geometrical perturbations in the unit cell of metasurfaces^18,22–26^. Recently, *q*BICs have been proposed and theoretically investigated^27–30^ and experimentally demonstrated^29^ in metasurfaces that are geometrically symmetric under an in-plane inversion, but permittivity asymmetric under the same spatial transformation (*C*_2_ or (*x*, *y*) → (−*x*, −*y*)).

Here we demonstrate that periodic permittivity changes act as a symmetry-breaking mechanism, enabling all-optical tailored resonances. These correspond to symmetry-protected permittivity-asymmetric *quasi*-bound states in the continuum (*ε*-*q*BICs), for which higher quality factors (*Q*) correlate with smaller permittivity asymmetries^27–30^, akin to GMRs^7–9^. But while GMRs are excited in hybrid grating/waveguide structures with predefined periodicities^31^, we demonstrate a higher control over *ε*-*q*BICs (in wavelength, *Q*, and amplitude) in an all-optical manner without any in-plane geometrical constraints.

**Fig. 1:**
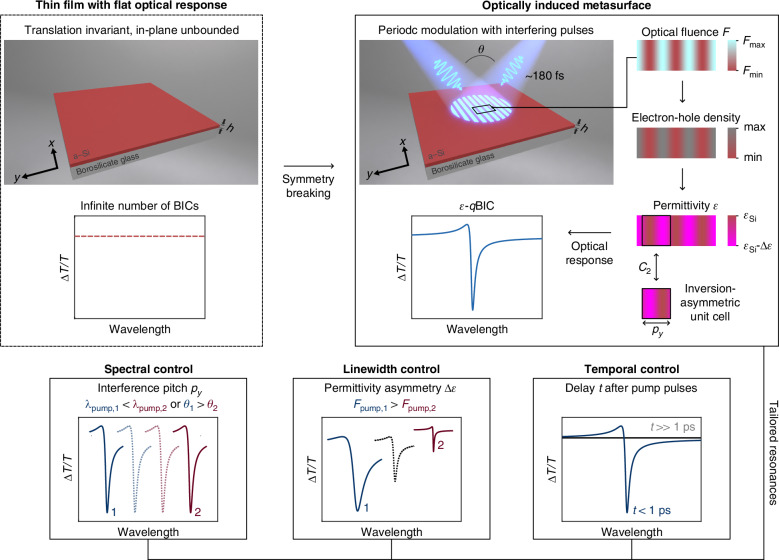
**Permittivity symmetry breaking of continuous media and tailored** ***ε*-*q*BICs**. (top left) An unbounded thin film of continuous translational symmetry features an infinite number of symmetry-protected BICs states (as possible localized electromagnetic field distributions in the film), which do not couple to a far-field impinging radiation (flat optical response). The wavelength of a particular BIC is then defined by the film thickness and unperturbed permittivity, and also by the dimension of the asymmetric illumination pattern. (top right) Scheme of the symmetry breaking through the interference of two ≈180 fs pulses on the a-Si thin film (of height *h*) deposited on a borosilicate cover glass. The pump beams interfere at an angle *θ* generating a 1D grating pattern of bright (BF) and dark fringes (DF) that excites electron-hole pairs (*e*^−^/*h*^+^) mainly at the BFs via absorption (middle panel). These excited carriers impose a periodicity *p*_*y*_ in the film permittivity (bottom panel), whose 1D unit cell is asymmetric under an in-plane inversion (*C*_2_ or (*x*, *y*) → (−*x*, −*y*)) transformation, considering how the electric field is distributed on the defined unit cell (Fig. 2d). The optically induced metasurface is able to temporarily sustain an *ε*-*q*BIC (Fano-shaped optical response). The dynamics of the *ε*-*q*BIC are relevant solely after the symmetry is broken and are independent of the prior existence of only its respective BIC state in the unperturbed film. Note that, although one may shift the proposed unit cell by half of its period and make it symmetric in its permittivity under an in-plane transformation (as for a metasurface of individual resonators in the absence of a superlattice^29^), the distribution of electric dipoles and fields in it (Fig. 2d) still leads to a non-zero coupling amplitude and to the emergence of a *quasi*-BIC^29^. Equivalently, the arbitrary definition of a new asymmetric unit cell from an initially permittivity- and geometrically-symmetric metasurface unit cell^18,29^ using an in-plane translation preserves the BIC state and a zero coupling amplitude. (bottom) Tailored resonances can be induced in the unpatterned film by changing different parameters of the pump beams

In a similar fashion to dynamic gratings, we are able to induce a permittivity symmetry breaking in an in-plane unbounded homogeneous medium by interfering two ≈180 fs ultrashort laser pulses on a thin film (Fig. 1, and Fig. S1 of the Supplementary Information—SI—for the optical setup used in the pump-probe experiments). Conversely to dynamic gratings, we probe the induced permittivity modulation at wavelengths longer than the induced periodicity, for which no diffraction effect is expected (other than those at zero order)^8,11^. Laser beam interference has been used for imaginary refractive index-symmetry breaking and ultrafast laser output control in perovskite metasurfaces, albeit with a resonance wavelength predefined by the fabricated array of holes^32^. Here, we periodically modulate the real part of the permittivity of an unstructured amorphous silicon (a-Si) film in space via a preferential carrier excitation in the bright fringes of the generated interference pattern (see Fig. S3 of the SI)^33^. This modulation breaks the original continuous translational symmetry of the system and imposes a periodic 1D unit cell that is asymmetric under in-plane inversion (Fig. 1 top right) due to the periodically modulated carrier distribution. The set of these periodic cells forms an optically induced metasurface, allowing the film to sustain *ε*-*q*BICs. The corresponding symmetry-protected BIC, i.e., the limiting case of unperturbed fringes, can be regarded as an electromagnetic mode exponentially confined in the *z*-direction, which varies sinusoidally in the *y*-direction and remains constant along the *x*-direction. This mode can be understood as the analog of a symmetry-protected BIC in an array of identical rod resonators^18,29^, whose in-plane (*xy*) gaps have been shrunk to zero. Note that the periodicities in a symmetric metasurface whose unit cell is comprised of equally spaced bars (i.e., it may be shifted by its pitch and half of its pitch in *y*) also exist in the unperturbed film. Thus, the symmetry of the out-of-phase dipoles in adjacent (yet to be formed) fringes in the film generates a perfect destructive interference in the far field, protecting the mode from radiative coupling. Employing this continuous, in-plane unbounded medium, we demonstrate significant optical modulation at normal incidence and ultrafast enhancement of nonlinear effects at selected wavelengths.

To achieve symmetry breaking, modulations in the permittivity of a thin film need to be induced locally by the pump beams. These perturbations can arise from a range of processes, including the Kerr effect, thermal expansion, molecular reorientation, and photorefraction. Some of the most significant changes can however be introduced by changing the electronic energy states, i.e., generating optically pumped free electrons and holes in a high-index semiconductor. a-Si is a suitable material for optical excitation and can be deposited in thin films with standard equipment. To prove the feasibility of the metasurface, a 45 nm thick a-Si film was deposited on a borosilicate glass substrate (Fig. 1, see “Methods” for fabrication details). The optically induced permittivity modulation on it is then produced by interfering two ultrafast (≈180 fs) laser pulses at an angle *θ*, which produces bright and dark interference fringes with a pitch along the *y*-axis related to the pump wavelength *λ*_*pump*_ via (see eq. 2.10 of ref. ^11^):

1 $${p}_{y}=\frac{{\lambda }_{pump}}{2\sin \left(\theta /2\right)}$$

To reach regimes with significant changes in the film’s permittivity, these pulses with energies up to 130 nJ are focused to a 1/*e*^2^ waist radius of 25 μm. Although the in-plane inversion symmetry might be broken with a single pump beam at an angle, this scheme does not provide the periodicity required by the *ε*-*q*BIC.

## Results

### Measuring all-optical *ε*-*q*BICs

**Fig. 2:**
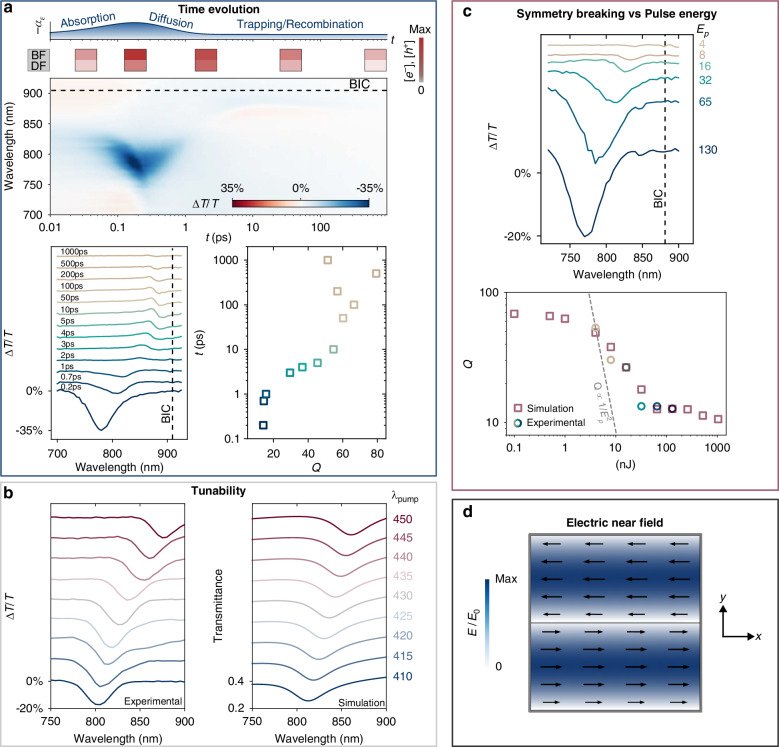
**Experimental all-optical** ***ε*-*q*BICs in a featureless thin film**. **a** Temporal evolution of the *ε*-*q*BIC from a 45 nm thick a-Si film measured at 130 nJ of pulse energy per arm (*λ*_*pump*_ = 420 nm, the smooth response of the film without a metasurface—orthogonal pump polarizations—has been subtracted from the signal for clarity). Schemes on top of the differential transmittance map depict *−α*_*ε*_ and the free-carrier concentration ([*e*^−^], [*h*^+^]) of bright (BF) and dark fringes (DF) at different times. The resonance red-shifts towards the symmetry-protected BIC state (horizontal—on the top panel, and vertical—on the bottom left panel, dashed lines, arbitrarily positioned for guiding purposes) for longer times. The bottom right panel shows *Q* vs *t*. *Q*-factors increase as the permittivity symmetry is restored (*α*_*ε*_ → 0) and losses are reduced due to carrier recombination. **b** Tunability of *ε*-*q*BICs as a function of *λ*_*pump*_. (left) Spectra measured at *θ* = 58° and 40 nJ laser pulse energy per arm. (right) Corresponding spectra obtained from simulations by varying *p*_*y*_ of the metasurface unit cell (corresponding *λ*_*pump*_ shown on the right). Parameters ${\varepsilon }_{r,{E}_{p}\to \infty }=$ 0.6, ${\varepsilon }_{i}^{M}=$ 0.25, ${\varepsilon }_{i,{E}_{p}\to \infty }=$ 1.15, *k*_*b*_ = 0.07 nJ^−1^, *k*_*d*_ = 0.002 nJ^−1^, and *E*_*p*_ = 40 nJ as defined in Eq. (2) were used in all simulations. See Fig. S15 of the SI for the permittivity model as a function of *E*_*p*_. **c** Symmetry breaking versus pump pulse energy per arm *E*_*p*_ (in nJ, shown on the right, *λ*_*pump*_ = 420 nm, see Fig. S8 for full differential transmittance maps). (bottom) Experimental (circles) and FEM-calculated (red squares) *Q*s vs *E*_*p*_. An inverse-square dependence ($Q\propto 1/{E}_{p}^{2}$) is shown as a dashed gray line. FEM calculations employed identical parameters used in simulations of the spectra shown in **b**. **d** Electric near-field polarization (arrows) and electric near-field enhancement (*E*/*E*_0_) of the *quasi*-symmetry-protected resonance. A 400 nm-wide section of the metasurface along the *x*-axis is shown. Note that the electric field vectors located in regions of different permittivities (upper and lower halves of the unit cell) point in opposite directions, and due to their different magnitudes, correspond to a *C*_2_ asymmetric configuration, as the permittivity does

Since the resonances appear isolated in time after the pump pulses arrive on the substrate, the induced transmittance changes are measured with synchronized white light pulses at variable time delays. By subtracting the intrinsically flat response of the a-Si film, it is possible to spectrally resolve the time dynamics of only the induced resonance. As seen in Fig. 2a, when the white light pulse is spatially and temporally superimposed with the grating, a significant (>30%) reduction of differential transmittance (Δ*T*/*T*) is observed in an isolated wavelength range, which correlates to the simulated response of an induced *ε*-*q*BIC under strong permittivity asymmetry (see below). This strong wavelength dependence is not expected from the theory of thin dynamic gratings^11^.

Changing the time delay of the probe pulses reveals a rich dynamic in the temporal evolution of the *ε*-*q*BIC, related to the carrier dynamics in the film. The induced resonance, at first, builds up during the first 200 fs (blue-shifting to ≈780 nm) as the pump pulses are absorbed mainly at the bright fringes, progressively breaking the permittivity symmetry of the film. This resonance shows a transmittance modulation of >30% for times 0.1 ps < *t* < 0.3 ps (see Fig. S4 of the SI), until it slightly red-shifts and mostly disappears within ~1 ps. Sub-ps dynamics has been previously reported on high carrier densities in bulk crystalline Si generated via pulsed interference patterns at small angles (implying an optical grating with a very large pitch, ≈13 μm)^33^. However, relaxation times of the optical response, due to hot carriers' relaxation to the band edges, had an opposite trend with pulse energy than we have observed (see below), and without any signs of strong wavelength dependence or attribution to resonant phenomena.

The decay shown in Fig. 2a is attributed to the migration of excited carriers from the bright to the dark fringes, as this ≈1 ps dynamic is too fast for a full carrier recombination in the thin film (see, for example, measurements of the carrier dynamics without the metasurface in Fig. S5 of the SI)^34^. This carrier migration, in turn, reduces the permittivity asymmetry of the unit cell, quantified by the asymmetry parameter ${\alpha }_{\varepsilon}\mathop{=}\limits^{{\rm{def}}}\Delta \varepsilon /\varepsilon$. As the redshift continues towards the symmetry-protected BIC state (horizontal dashed line in Fig. 2a top—defined by *p*_*y*_, the substrate, the film height, and unperturbed permittivity), also due to an increased refractive index attributed to carrier recombination, a second spectral feature appears. At *t* ≈ 4 ps, a long-lived resonance emerges that lasts for >1000 ps, attributed to trapped carriers at the bright fringes that sustain a small permittivity asymmetry (implying *α*_*ε*_ ≠ 0) in the metasurface. The spectra (cross-sections of the top panel) and the corresponding *Q*-factors at different times are shown in the bottom left and bottom right panels in Fig. 2a, respectively. Experimental *Q*-factors were obtained through a fitting procedure using a Fano-shaped function as described in the caption of Fig. S12 of the Supplementary Information.

A few aspects play a role in these dynamics. As mentioned, the excited carriers diffuse from the bright to the dark fringes, reducing the permittivity asymmetry of the system. Smaller values of *α*_*ε*_ shift the resonance closer to the BIC state and are correlated with higher *Q*-factors^29^. Besides, the excited free electrons and holes recombine and the film becomes less lossy, being able to sustain higher-*Q* *ε*-*q*BICs. This explains the appearance and redshift of the second spectral feature observed in the differential transmittance measurements, and the respective increase in *Q* for longer times, as shown in Fig. 2a (bottom right).

To prove the spectral tunability of the metasurface, measurements were performed at different pump wavelengths, leading to different periodicities *p*_*y*_ of the permittivity modulation (see Eq. (1)). The spectral response at the highest modulation for each *λ*_*pump*_ is shown in Fig. 2b, along with their respective numerically calculated spectra. By red-shifting the pump wavelength from 410 to 450 nm with a fixed interference angle *θ* = 58°, we observe a redshift of the resonance from 805 to 875 nm (see Fig. S6 of the SI for the full differential transmittance maps). By only varying the periodicity in simulations we are able to reproduce numerically the experimental spectra in Fig. 2b. The optical responses can then be calculated, with good agreement, using solely the unit cell size of the metasurface and a modified complex permittivity of a-Si as a function of the employed pump pulse energy (see below and the Numerical calculations in “Methods” for details). We are here neglecting the different carrier excitation efficiencies between the pump wavelengths in the formation of the metasurface.

In addition to this spectral tunability, we expect that different asymmetries lead to different *Q*-factors of the resonance. In this system, the asymmetry can be continuously adjusted by changing the incident pump pulse energy, which however also introduces different losses and changes the overall refractive index of the medium. To evaluate the response of the film and, thus, the behavior of resonances, we have therefore measured the transmission spectra at different pump pulse energies (*E*_*p*_) between 4 and 130 nJ. As seen in Fig. 2c (top), higher *E*_*p*_ values blue-shift the resonance away from the true BIC state (numerically calculated vertical dashed line). The slight deviations in *ε*-*q*BIC wavelength and transmittance modulation from Fig. 2a are due to small differences in optical system alignment and the sensitivity of the BIC to the interference angle *θ* (Eq. (1)). Additionally, slower decay times are observed for lower pump energies, due to the interplay between a slower carrier diffusion and lower losses due to an overall smaller excited carrier density (see the complete differential transmittance maps of the *ε*-*q*BICs as a function of the pump pulse energy in Fig. S8 of the SI). A similar trend is observed for a pump wavelength of 410 nm (Figs. S9 and S10 of the SI). Therefore, by merely changing the pump pulse energy not only the wavelength and amplitude of resonances can be modified, but also their *Q*-factor and decay times.

A hallmark of symmetry-protected *q*BICs is the inverse-square dependence of the *Q*-factor with the asymmetry parameter *α*, as derived from a perturbative treatment of the BIC state^18,29^. This behavior occurs for a system in which the losses are dominated by radiative processes (i.e., photon emission through coupling to the continuum of states induced by geometrical/permittivity asymmetries). This implies that either non-radiative losses (absorption) are completely absent, which is an ideal case, or can be neglected when compared to radiative losses. If the *Q*-factor is described by a reciprocal sum of radiative (*r*) and non-radiative (*nr*) quality factors (1/*Q* = 1/*Q*_*r*_ + 1/*Q*_*nr*_), *Q*_*nr*_ → *∞*, and thus *Q* = *Q*_*r*_. As the pump pulse energy is our symmetry-breaking tuning parameter, it is worth investigating how *Q* behaves as a function of *E*_*p*_, and if *E*_*p*_ can play an analogous role of geometrical^18^ or in-plane momentum perturbations^35^ in symmetry-protected *q*BICs (i.e., $Q\propto 1/{E}_{p}^{2}$).

In the bottom panel of Fig. 2c we show the calculated (red squares) and the experimental (circles) values of *Q* as a function of *E*_*p*_. The calculations for the ideal lossless metasurface versus −*α*_*ε*_ is shown in Fig. S11 of the SI. As expected, when dealing with a system devoid of intrinsic losses *Q* of the metasurface follows an inverse-square dependence with *α*_*ε*_ ($Q\propto {\alpha }_{\varepsilon }^{2}$), in agreement with theoretical predictions^28–30^. This corresponds to a situation where the permittivity difference between the bright and dark fringes in the 1D unit cell is gradually increased, and the losses in the grating only occur due to radiative processes as the metasurface resonance progressively couples to propagating states in the continuum. Note that *Q* > 10^11^ is in principle possible in the absence of non-radiative losses (Fig. S11). When the experimental *Q* (see Fig. S12 for fitted data) is plotted against *E*_*p*_, however, an obvious deviation from a $1/{E}_{p}^{2}$ behavior (dashed gray line) is observed, which implies that losses are present and the asymmetry parameter *α*_*ε*_ is not a mere linear function of *E*_*p*_ in the measured range. In spite of these deviations, we may reproduce the observed behavior by resorting to a simple model of the permittivity of bright and dark fringes as a function of *E*_*p*_. We may model the dispersive permittivity of the thin Si film (${\varepsilon }_{b(d)}^{Si}(\omega ,{E}_{p})$) in the bright (dark) fringes as a function of the pump pulse energy *E*_*p*_ as:

2 $$\begin{array}{ll}{\varepsilon }_{b(d)}^{Si}(\omega ,{E}_{p})\,=\,{\varepsilon }_{r}^{Si}(\omega )\times \left[{\varepsilon }_{r,\,{E}_{p}\to \infty }+(1-{\varepsilon }_{r,{E}_{p}\to \infty }){e}^{-{k}_{b(d)}{E}_{p}}\right]\\\qquad\qquad\qquad+\,i\left[{\varepsilon }_{i}^{Si}(\omega )+{\varepsilon }_{i}^{M}+{\varepsilon }_{i,\,{E}_{p}\to \infty }(1-{e}^{-{k}_{b(d)}{E}_{p}})\right]\end{array}$$

where ${\varepsilon }_{r}^{Si}(\omega )$ and ${\varepsilon }_{i}^{Si}(\omega )$ are the real and the imaginary permittivities, respectively, of the unperturbed a-Si film as measured via ellipsometry (Fig. S14 of the SI), ${\varepsilon }_{i}^{M}$ a parameter that accounts for losses due to the finite size of the metasurface, ${\varepsilon }_{r,{E}_{p}\to \infty }$ the real (fractional) and ${\varepsilon }_{i,{E}_{p}\to \infty }$ the (additive) imaginary saturation values of the permittivity for *E*_*p*_ → *∞*, and *k*_*b*(*d*)_ a constant that defines the exponential changes in the bright (dark) fringes. Three important aspects are represented in this model: (i) At very small pulse energies, *E*_*p*_ → 0, losses are limited by the imaginary permittivity of the film and the metasurface size (${\mathfrak{Im}}({\varepsilon }_{b(d)}^{Si}(\omega ,{E}_{p}\to 0))\approx {\varepsilon }_{i}^{Si}(\omega )+{\varepsilon }_{i}^{M}$); (ii) at low pulse energies, a linear change in the permittivity is obtained (${e}^{-{k}_{b(d)}{E}_{p}}\approx 1-{k}_{b(d)}{E}_{p}$); (iii) at high pulse energies, *E*_*p*_ → *∞*, a saturation regime is achieved (${\varepsilon }_{b(d)}^{Si}(\omega ,{E}_{p}\to \infty )={\varepsilon }_{r}^{Si}(\omega )\times {\varepsilon}_{r,{E}_{p}\to \infty }+i({\varepsilon }_{i}^{Si}(\omega )+{\varepsilon }_{i}^{M}+{\varepsilon}_{i,{E}_{p}\to \infty})$, Fig. S15 of the SI).

This simple model is able to reproduce the trend in the measured *Q*s with a fairly good agreement. Counterintuitively, the model predicts a reduction in the asymmetry parameter (*α*_*ε*_) for higher pump pulse energies, as the bright fringe optical response to the pump beams saturates while the dark fringe permittivity is further reduced with increasing pump absorption (Fig. S15 of the SI). In addition, the model predicts a saturation of *Q* at low pulse energies (*Q* ≈ 70, where losses are dominated by the finiteness of the metasurface, ${\varepsilon }_{i}^{M}$, chosen to be equal to 0.25 to better reproduce the experimental data). Although changing *E*_*p*_ allows us to navigate through the parameter space of asymmetries, assessing the system’s response to minute values of *α*_*ε*_ and probing an eventual $Q\propto 1/{E}_{p}^{2}$ dependence requires the generation of larger metasurfaces than we are currently able with our optical setup. Besides, as the pump pulses have a Gaussian-shaped intensity distribution from its center, different carrier densities are excited at different positions in the optical 1D grating, leading to different permittivity asymmetries as we move from the center of the grating towards its edge. Naturally, by representing the metasurface using the unit cell only we are neglecting this spatial variation of *α*_*ε*_, nonetheless being able to reproduce the observed experimental behavior (see Fig. S16 of the SI for the spectral response as a function of *E*_*p*_). If the imaginary part of the permittivity of the fringes is arbitrarily reduced for a given *E*_*p*_, a second, spatially-orthogonal, resonance appears, which corresponds to the GMR and is less resilient to optical losses (see Fig. S17). However, for losses experienced at common experimental conditions, only the *ε*-*q*BIC is observed as exemplified by its characteristic near-field distribution (Fig. S17). This behavior of neighboring symmetry-protected *q*BIC and GMR modes has previously been observed in nanostructured 1D gratings^36^. Further characterization of the all-optical *ε*-*q*BICs (pump and probe polarization dependence, etc) can be found in Figs. S18–S21 of the SI.

### Spectrally selective third-harmonic generation enhancement via *ε*-*q*BICs

*ε*-*q*BICs excited via optically induced metasurfaces are expected to carry the same functionalities of resonator-based metasurfaces. One of the most utilized properties of metasurfaces featuring resonances is their optical near-field enhancement, which can be used to boost phenomena that require strong light-matter interaction, like nonlinear effects^36,37^. Symmetry (centrosymmetry) breaking via DC electric fields has been long used to induce second-harmonic generation in centrosymmetric materials^38^, although it is not a spectrally selective method. As a proof-of-principle, we demonstrate how the induced in-plane symmetry breaking can be used to enhance the third-harmonic generation (THG) at specific wavelengths in a bare silicon film within a narrow timeframe.

**Fig. 3:**
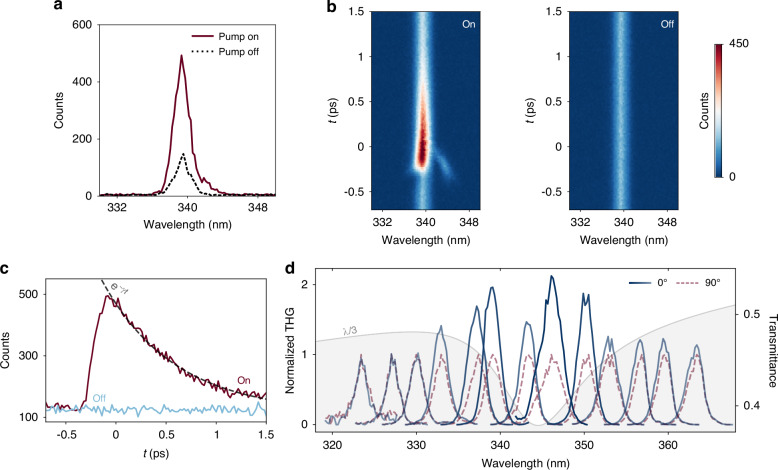
**Spectrally selective third-harmonic generation enhancement using** ***ε*-*q*BICs**. **a** THG signal (*E*_*p*_ = 75 nJ) in the presence (pump on) and absence (pump off) of the optical grating (*λ*_*pump*_ = 515 nm) providing the permittivity symmetry breaking. A 234% enhancement of THG (at 340 nm, *λ*_*fundamental*_ = 1020 nm) is obtained when a metasurface is generated in the thin film (solid red line) compared to when both pump beams are blocked (dashed black). **b** THG signal vs delay line position, whose cross-sections in time are shown in **(a)**. *t* = 0 is defined near the maximum of the THG signal. **c** Corresponding THG signals as a function of time at 339 nm. The enhancement provided by the *ε*-*q*BIC decays exponentially (1/*e* ≈ 0.72 ps, fit shown as a gray dashed line). See Fig. S22 for full data. **d** Normalized THG signal for 0° (blue curves) and 90° (dashed light red) relative pump polarizations as a function of the fundamental beam wavelength (from *λ*_*fundamental*_ = 970 nm to *λ*_*fundamental*_ = 1090 nm). A preferential THG enhancement is observed when the fundamental is overlapped with the *ε*-*q*BIC (transmittance shown as a gray shadowed area, right-hand side axis). Parameters ${\varepsilon }_{r,{E}_{p}\to \infty }=$ 0.75, ${\varepsilon }_{i}^{M}=$ 0.25, ${\varepsilon }_{i,{E}_{p}\to \infty }=$ 1.05, *k*_*b*_ = 0.06 nJ^−1^, *k*_*d*_ = 0.002 nJ^−1^, *E*_*p*_ = 50 nJ and *θ* = 51° (implying *p*_*y*_ = 598 nm) were used in the transmittance calculation. A >100% enhancement is obtained for *λ*_*fundamental*_ = 1040 nm. *ε*-*q*BIC spectrum was shifted to a third of its value (*λ*/3) to overlap with THG signals

For that, we generate a grating at a 515 nm pump, which we now probe with pulses of tunable narrow bandwidth (fundamental beam) instead of the supercontinuum white light. The longer pump wavelength increases the *p*_*y*_ of the metasurface, red-shifting the *ε*-*q*BIC further into the infrared range, and therefore its THG closer to the visible. In Fig. 3a the THG response of the bare film around 340 nm (*λ*_*fundamental*_ = 1020 nm) is compared when both pump beams are either turned on (solid red) or turned off (dashed black). A 234% enhancement of the THG signal is obtained when the pump beams illuminate the sample and break the in-plane inversion permittivity symmetry of the thin film. The corresponding THG signals as a function of time are shown in Fig. 3b, c. The spot at ≈343 nm in Fig. 3b is due to a four-wave mixing process, disappearing in the absence of the pump beams (Fig. 3b right, see SI for further characterization). The decay of the THG enhancement clearly follows an exponential trend (fitted gray dashed line) instead of a Gaussian temporal profile expected from an enhancement due to a mixing of ultrafast beams. This ≈1 ps dynamic is temporally similar to the excitation of *ε*-*q*BICs as shown in Fig. 2a. Although higher *Q*-factors are observed at longer times due to carrier recombination, the reduction in the THG enhancement is attributed to a smaller coupling efficiency of the fundamental beam to the finite-sized, lossy metasurface. This can be observed in the smaller modulation of the probe beam at such times (Fig. 2a).

To evaluate the spectral response of the nonlinear effect sweeps at different probe wavelengths were taken and referenced with sweeps where the pump pulses were cross-polarized, which switched off the grating contrast. Given the different collection efficiencies at different wavelengths of our optical setup, we have normalized the THG output (see Figs. S26–S28 in the SI for the raw data). By comparing the THG signals with (0°) and without (90°) the metasurface, we observed a preferential enhancement for probe wavelengths that are spectrally overlapped with the *ε*-*q*BIC (gray shadowed area in Fig. 3d, whose spectra were shifted to 1/3 of its value to overlap with the THG output). Here, a >100% enhancement was obtained for *λ*_*fundamental*_ = 1040 nm (*λ*_*THG*_ ≈ 347 nm) in the presence of the metasurface, when compared to the 90°-rotated polarization of one of the pump beams, despite all incident radiation of pump and probe having the same pulse energy in both situations. The lower enhancement is attributed to the smaller pump energy used for this set of measurements (*E*_*p*_ = 50 nJ vs *E*_*p*_ = 75 nJ in Fig. 3a).

## Discussion

We have shown that a simple unpatterned dielectric amorphous film can yield tailored resonances when its permittivity is periodically modulated in space, breaking its original translational and in-plane inversion symmetries. Optical resonances do not require then to be predefined and constrained by etching and deposition fabrication procedures that provide the boundary conditions for typical spatially-finite resonators. *ε*-*q*BICs demonstrated here in the near-infrared can easily be extended further into the visible and towards the mid- and far-infrared, where coupling to excitonic resonances^39^ and molecular vibrational/rotational levels could be performed^40,41^.

Periodic permittivity variations in space are also a core concept in distributed Bragg reflectors (DBRs) and photonic crystals (PhCs)^42–44^. But, conversely to DBRs and PhCs, where confinement of light deteriorates with a smaller contrast of refractive indices, here higher localization and, thus, higher *Q*-factors are achieved with smaller spatial permittivity differences. This facilitates the dynamic modulation of devices, as it reduces—or, in principle, completely circumvents—the need for large alterations in optical properties of materials with a given input to significantly change their optical response. The minute values of electro-optical coefficients of most materials here play to our advantage, and a number of substances which were previously dismissed as “optically inert" can now be incorporated into the set of electro-optical materials, including silicon. Besides free carriers, any permittivity-change mechanism can be employed for symmetry breaking and the excitation of *ε*-*q*BICs in a thin film, be it via linear (Pockels)^45^ or nonlinear (Kerr) electric-field effects^46^, ion implantation^47^, temperature differences^16^, etc.

The broad optical responses in condensed matter imply that any given input aimed to modulate the permittivity of materials would also affect it in a broadband fashion^2,48^. For this reason, optical signals that are encoded in different frequencies within an optical fiber (wavelength-division multiplexing) need to be physically separated in demultiplexing operations. By leveraging the strong and spectrally localized resonances that arise from small perturbations in permittivity symmetries in an otherwise optically flat platform, we may modulate/filter the propagation of a small set of wavelengths independently, leaving the propagation of other frequencies nearly undisturbed. Selective optical modulation using *ε*-*q*BICs across all optical telecommunication bands (from the high-energy edge of the O band at 1260 nm to the low-energy end of the L band at 1625 nm) can, in principle, be easily performed in the same thin silicon film.

As we have demonstrated, several degrees of freedom can be used to tailor the desired resonances, such as pump pulse energy and wavelength, relative delay, incidence angle, and polarization of the grating-forming beams. Another advantage of a homogeneous medium relative to pre-fabricated metasurfaces of resonators is the absence of losses due to corners, edges, and fabrication imperfections. This makes defect-free crystalline films a sort of ultimate platform for on-demand high-*Q* modes. We have shown the excitation of *ε*-*q*BICs through a rather lossy symmetry-breaking mechanism, although it allowed us to exploit large values of the asymmetry parameter *α*_*ε*_ and a way to assess ultrafast carrier dynamics and spatial distribution in solid state systems^11^. Besides, selectively enhancing nonlinearities in a platform whose frequency-conversion efficiency is not constrained by phase-matching requirements, but instead governed by the induced resonances, provides great flexibility when exploiting nonlinear phenomena. Crucial questions concern what practical limits this platform can achieve, and what other asymmetries may be exploited^27,28,32^. The features of active metasurfaces may be expanded to include permittivity symmetry breaking in continuous media.

## Conclusion

In conclusion, we have demonstrated that an unbounded, featureless thin film with an intrinsically flat optical response can sustain resonances at desired wavelengths, amplitudes, and *Q*-factors when a particular permittivity asymmetry is induced in it via optical means, i.e., by interfering two ultrafast laser beams at an angle. We have argued that the nontrivial emergence of strong, frequency-dependent, optical responses follows directly from the symmetry breaking of the original continuous translational and in-plane inversion permittivity symmetries of the film.

Experiments were performed in one of the simplest CMOS-compatible platforms, namely, an amorphous Si (a-Si) film over borosilicate glass. Transmittance modulations as high as 35% and *Q* ≈ 80 for long-lasting resonances (observed due to carrier trapping) were achieved via carrier excitation in the semiconductor, an inherently lossy symmetry-breaking mechanism. Significant improvements are expected in modulation amplitudes and in *Q*-factors of *ε*-*q*BICs if a crystalline Si film, inherently less lossy, is used instead, by pumping at longer wavelengths and also by exploiting larger metasurfaces than we have used here. For that, pico- and nanosecond long pump pulses could be used due to the required temporal and spatial overlap between them. We have also demonstrated a sub-ps > 200% enhancement of THG in the bare Si film at wavelengths corresponding to the *ε*-*q*BIC excitation. Again, the inherently lossy mechanism of symmetry breaking due to carrier excitation inhibits larger enhancements of the third harmonic than we have observed, a process whose efficiency should scale enormously for larger *Qs* (proportional to the dwelling time of the photons in the film) and due to increasing near-field enhancement.

We believe that *ε*-*q*BICs in featureless continuous media have a strong connection with GMRs in unpatterned thin films^7–9^. Indeed, a *Q* ∝ 1/*α*^2^(*θ*) dependence and the symmetry-protected nature of GMRs have been observed in silicon cuboid metasurfaces at THz frequencies due to an incidence angle(*θ*)-induced symmetry breaking^49^. However, while GMRs are essentially invariant in one dimension, all-optical *ε*-*q*BICs of higher dimensionality could be excited^27–30,32^. Besides, GMRs have required so far featureless thin films to be coupled to corrugated structures in resonant waveguide gratings^31^ or to bear inclusions in high-contrast gratings^50^, both already exploited in several applications. The addition of corrugations or inclusions, however, inevitably defines the periodicity of the structure and thus its resonant wavelength, limiting the spectral flexibility of the design. All-optical *ε*-*q*BICs here demonstrated do not have this structural limitation.

We hope that the all-optical excitation of *ε*-*q*BICs will provide new ways of dynamically manipulating electromagnetic radiation at required wavelengths in a single lithography-free platform and bring new possibilities for light technologies. These may encompass applications as varied as optical filters^8^, *Q*-switching^51^, holography, focusing and steering of optical beams^52^ and also thermal emission control^53^ through the interplay of local and nonlocal responses, chiral^54^ and orbital angular momentum^55^ manipulation, generation of second- and higher-order harmonics^36,37^, optical modulators^56^, tunable lasing^24,25,32,57^, sensing^41^, coupling to vibrational^40^ and excitonic resonances^39^ in controllable weak to ultrastrong light-matter interaction regimes^58^ and polaritonic condensation^59^.

## Methods

### Numerical calculations

Thin film spectra were calculated through finite-difference time-domain solutions of Maxwell’s equations using the commercially available software Lumerical (Ansys). A normal incidence, linearly-polarized (along the *x*-axis) plane wave propagating in the −*z* direction illuminated the thin film. Periodic boundary conditions were used to represent the in-plane (*xy*) unbounded media. Power monitors were used for transmittance calculations, and perfectly matched layers (PMLs) domains at the top and bottom for the absorption of propagating waves. A dispersionless refractive index of *n* = 1.45 was employed for the borosilicate substrate, while the dispersive complex permittivity of the amorphous silicon (a-Si), as measured via ellipsometry was used for the thin film (see Supplementary Information for data, Fig. S14). The dispersive complex permittivity of a-Si was then modified with the model proposed in Eq. (2) for bright and dark fringes (Fig. S15 of the SI). The modified permittivity was considered homogeneous in each fringe. The assumption of a constant *e*^−^/*h*^+^ plasma (and thus permittivity) along the film height is a good approximation for a 45-nm thin a-Si layer^34^. Eigenfrequency solutions of Maxwell’s equations were used for *Q*-factor calculations and were performed using the commercially available RF module of the finite element solver COMSOL Multiphysics with identical boundary conditions as described above. In both types of simulations, we have represented the optically induced metasurface using only the unit cell (Fig. 1, top right). This approximation reduces the computational resources required for simulating and predicting the desired optical responses of the thin film when compared to a simulation of the full grating domain (tens of μm × tens of μm). By simulating the unit cell alone we have then neglected spatial permittivity variations from the center towards the outer edges of the metasurface, which would be inevitably induced by the Gaussian intensity profiles of the pump beams used to generate the grating.

### Sample fabrication

a-Si was deposited onto borosilicate glass substrates via PECVD. Samples were annealed at 700 °C for 90 s to improve film quality.

### Optical measurements

A collinear optical parametric amplifier (OPA, ORPHEUS-HP) pumped by a pulsed Yb:KGW Pharos laser system (Light Conversion Ltd.) with a maximum repetition rate of 200 kHz generated outputs of pulse duration ≈180 fs. For time-dependent transmittance measurements, the 515 nm invariant beam output of the OPA was focused onto a 5-mm-thick sapphire plate and used to generate the supercontinuum probe light beam (see spectrum in the SI, Fig. S2), while the mechanically chopped wavelength tunable OPA output was sent through a 50/50 beam splitter to generate the metasurface. A motorized optical delay line was used to introduce controlled time differences between the pump and supercontinuum pulses. One of the pump beams passed through a controlled half-wave plate and a neutral density wheel for adjustable attenuation. The grating-generating pump beams and the supercontinuum light were slightly focused onto the sample using lenses of focal lengths equal to 100 and 75 mm, respectively. The pump beams have a spot size of ≈50 μm in 1/*e*^2^ diameter, while the probe beam has a spot diameter of ≈4 μm. Note that, due to the pump beams’ angle, the formation of the grating is not instantaneous, and the outer edges of the beams reach the thin film first, being absorbed without the grating formation. The generated free carriers decrease the film permittivity and increase its optical losses at these regions overall without any pattern being formed. These losses have a detrimental effect on the later induced resonances but do not play a significant role in the symmetry breaking nor in the conclusions we have drawn. Transmittance measurements were carried out with a lock-in detection system (Stanford Research Instruments) by modulating the wavelength tunable OPA output at <2 kHz frequency using an optical chopper. A spectrograph (PI Acton SP2300, Princeton Instruments) coupled to an avalanche photodiode (Thorlabs APD440A) was used for spectral characterization of the supercontinuum probe light transmitted by the sample. THG measurements were performed by using the invariant 515 nm output of the OPA to generate the metasurface, while the variable output (previously used as the pump beams) was used as the fundamental beam to generate the third-harmonic response in the thin film.

## Supplementary information

Supplementary Information for All-optical permittivity-asymmetric quasi-bound states in the Continuum

## Data Availability

The data that support the findings of this study are available from the corresponding author upon reasonable request.
