# Supplementary material for: All-optical permittivity-asymmetric quasi-bound states in the continuum
Source: Light Sci Appl. 2025 May 7;14:185. doi: 10.1038/s41377-025-01843-9 (PMC12059027; doi:10.1038/s41377-025-01843-9)
Supplement: Supplementary file 1 — Supplementary Information for All-optical permittivity-asymmetric quasi-bound states in the Continuum [file 41377_2025_1843_MOESM1_ESM.pdf]

Supplementary Information for

All-optical permittivity-asymmetric *quasi*-bound states in the continuum

Rodrigo Berté,[1,*] Thomas Possmayer,[1,*] Andreas Tittl,[1] Leonardo de S. Menezes,[1,2] and Stefan A. Maier[3,4]

[1] *Chair in Hybrid Nanosystems, Nanoinstitut München, Fakultät für Physik, Ludwig-Maximilians-Universität München, 80799 München, Germany*

[2] *Departamento de Física, Universidade Federal de Pernambuco, Recife, Pernambuco 50670-901, Brazil*

[3] *School of Physics and Astronomy, Monash University, Clayton, Victoria 3800, Australia*

[4] *The Blackett Laboratory, Department of Physics, Imperial College London, London SW7 2AZ, United Kingdom*

* Contributed equally to this work

OPTICAL SETUP

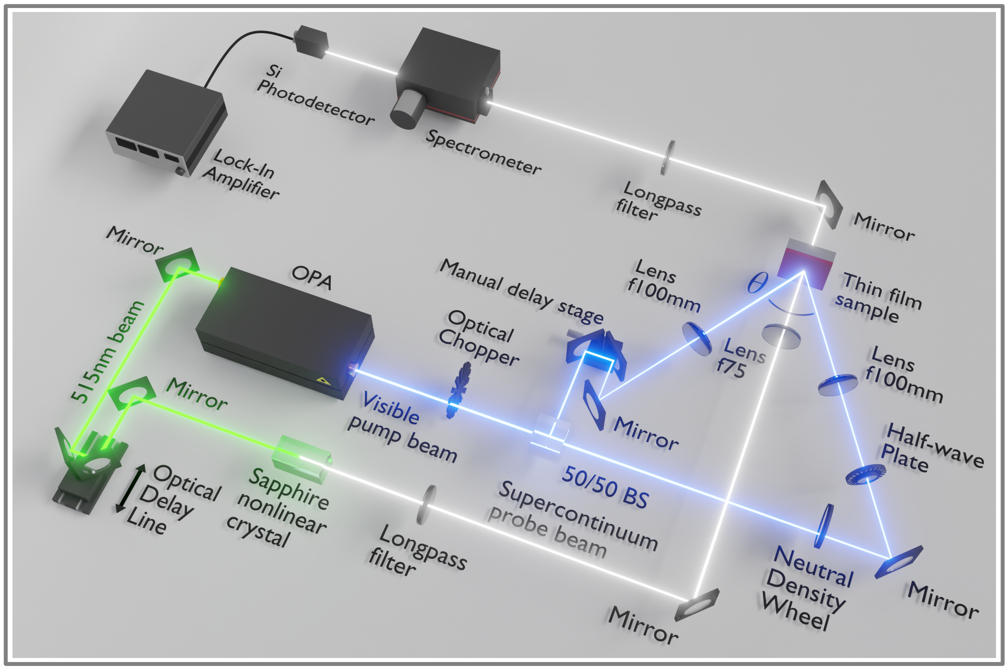

FIG. S1. Scheme of the optical setup employed in the generation of the optically-induced metasurface and detection of ε-qBICs. The manual delay stage (precision of ≈ 2 μm) is meant to compensate for the difference in optical path between the pump pulses, and not for a precise control between their relative phases. The effects of adjusting the manual delay stage are shown in Fig. S3. The spectrum of the supercontinuum probe beam is shown in Fig. S2.

SPECTRUM OF THE SUPERCONTINUUM PROBE BEAM

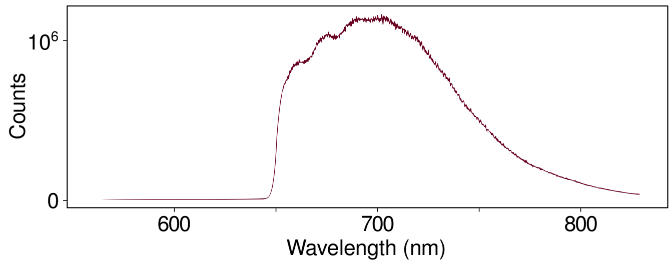

FIG. S2. Spectrum of the supercontinuum probe beam generated from the 515 nm OPA output ($P_{515nm} = 210$ mW) in a sapphire nonlinear crystal. The spectrum was filtered with a long-pass 650 nm optical filter prior to measurements. The generated supercontinuum beam is heavily chirped, which has been manually corrected in the post-processing of data for analysis when necessary.

OPTICAL RESPONSE VERSUS PATH (TIME) DELAY BETWEEN PUMP PULSES

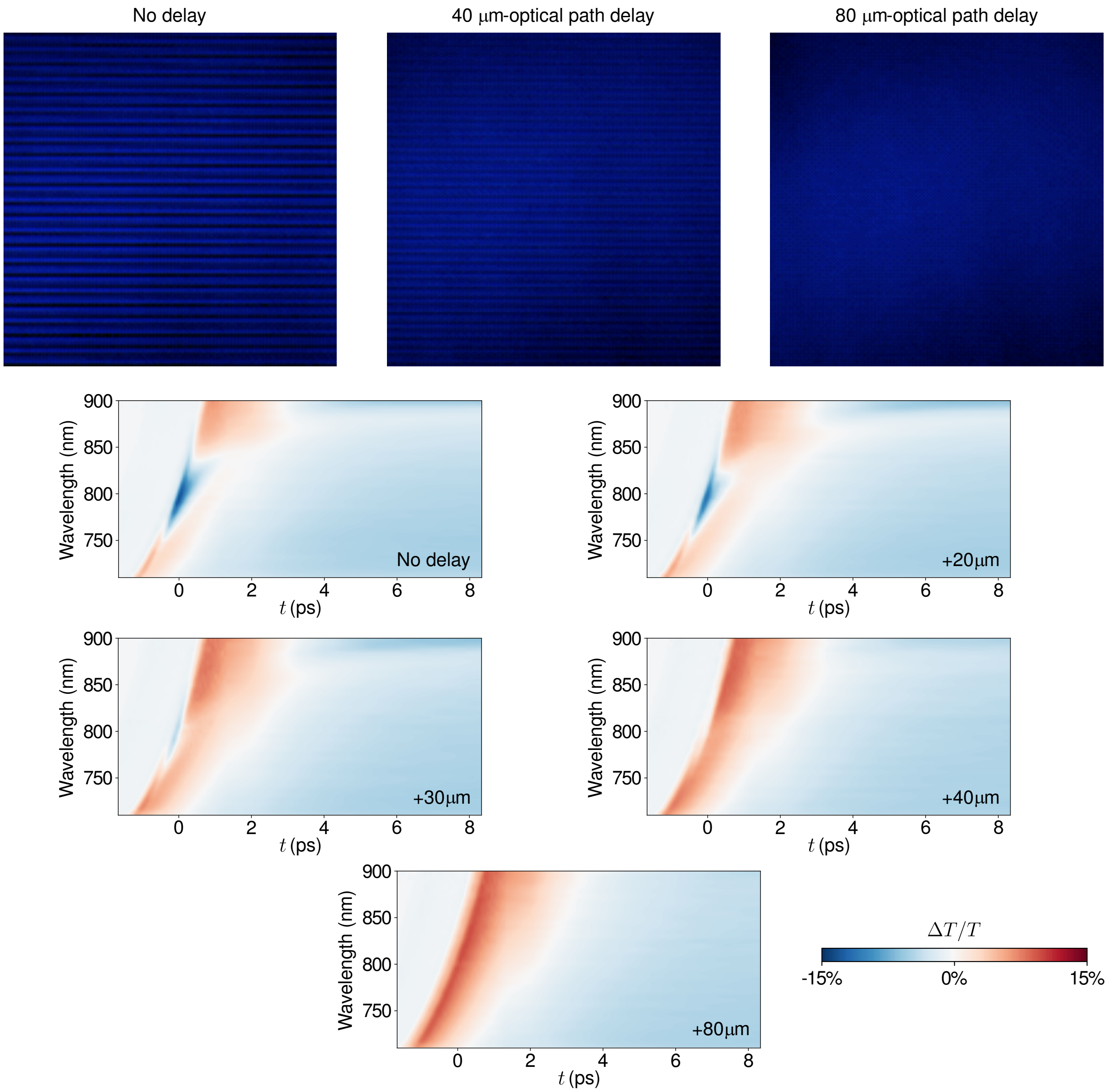

FIG. S3. Optical micrographs ($\lambda = 430nm$) without (left), and with temporal delays between pulses induced by a 40 μm (or 40 μm/3×10^8 m s^{-1} = 133 fs, center) and by an 80 μm (or 266 fs, right) increase in the optical path of one of the pump beams. The 1D optical grating progressively disappears as the pulses arrive in the thin film at increasingly different times. (bottom) Temporal evolution of ε-qBICs as a function of the optical path delay (shown in the insets). As the 1D optical grating disappears, so does the induced resonance, and only the flat optical response of the thin film is observed.

TEMPORAL CROSS SECTIONS OF ε-QBICS

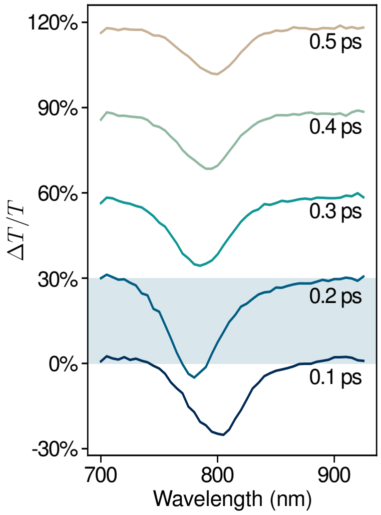

FIG. S4. Temporal cross-sections of data shown in Fig. 2a of the main manuscript, for 0.1 ps $< t <$ 0.5 ps. Although the main resonance lasts for $\approx$ 1 ps, the transmittance modulation is larger than 30% (shadowed area) only between 0.1 ps and 0.3 ps. The cross-sections at different probe delays are shifted by 30% for better visualization.

OPTICAL RESPONSE OF THE FILM VERSUS PUMP PULSE ENERGY IN THE ABSENCE OF THE OPTICALLY-INDUCED METASURFACE

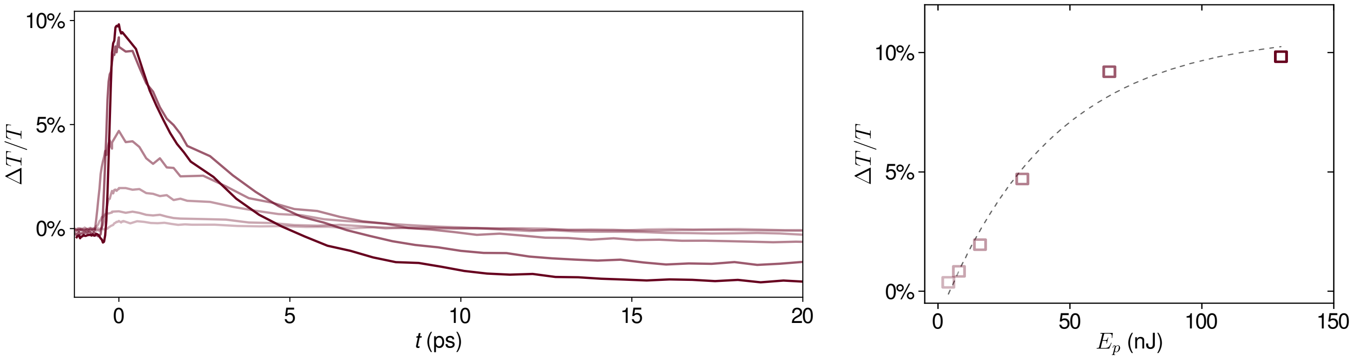

FIG. S5. (left) Differential transmissivity at 800 nm as a function of time for a 90°-rotated relative polarization between the pump beams (a condition where no 1D optical grating, thus no metasurface, is formed). Increasing pump pulse energies are shown in darker red colors. λ_{pump} = 420 nm. (right) Respective maximum differential transmissivity vs pump pulse energy per arm (at $t = 0$ ps). Dashed line shows the exponential fit ($\Delta T/T = a_0 + a_1 e^{-kE_p}$) of the data. The saturation of the optical response and a deviation from a linear behavior are clearly observed for pulse energies per arm larger than 65 nJ.

TEMPORAL EVOLUTION OF ε-QBICS AS A FUNCTION OF THE PUMP WAVELENGTH

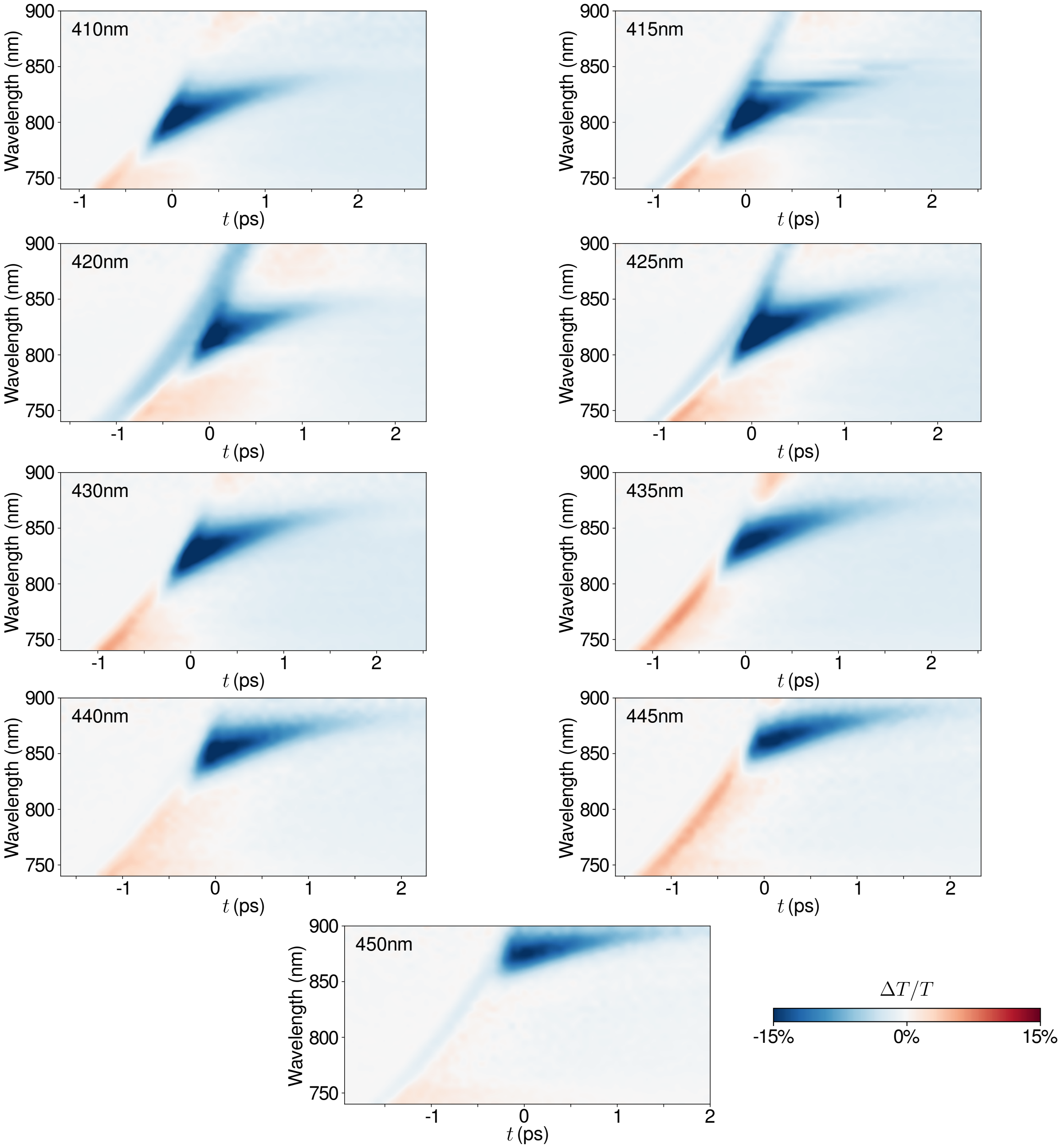

FIG. S6. Temporal evolution of ε-qBICs as a function of the pump wavelength (insets). Resonances red-shift for longer pump wavelenghts, corresponding to larger periodicities p_y of the metasurface. All resonances have a similar temporal profile, lasting for $\approx$ 1 ps and shifting towards the symmetry-protected BIC state for longer times, as the permittivity symmetry is restored due to carrier migration/recombination. Film response without the metasurface was subtracted to highlight resonances.

OPTICAL GRATING PERIODICITY VERSUS PUMP WAVELENGTH

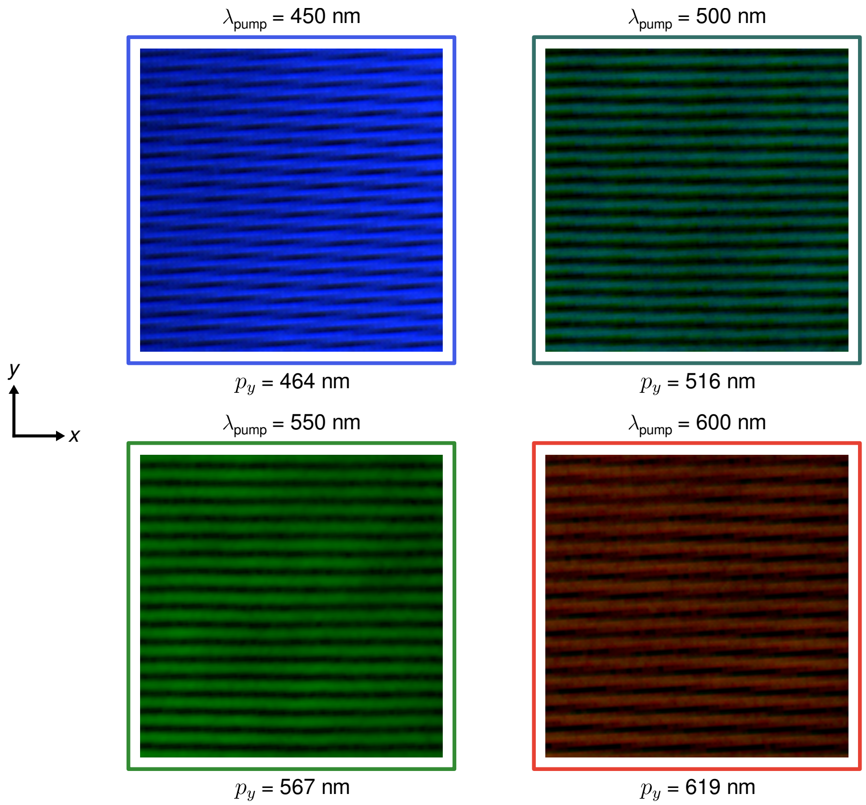

FIG. S7. Micrographs of optical 1DGs: larger periodicities (p_y) are notably formed for longer excitation wavelengths λ_{pump} for the same incidence angle $\theta = 58°$.

OPTICAL RESPONSE VERSUS THE PUMP PULSE ENERGY

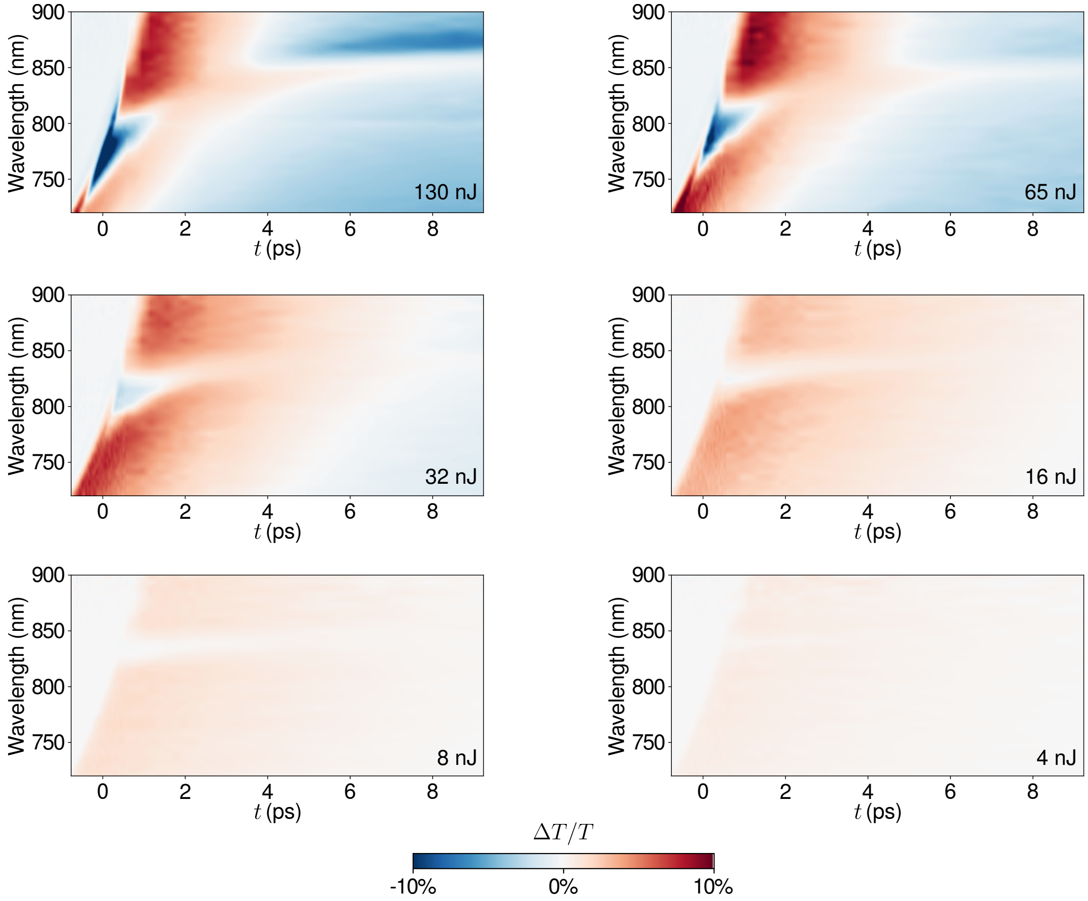

FIG. S8. Temporal evolution of ε-qBICs as a function of the pump pulse energy per arm (indicated in the insets), λ_{pump} = 420 nm. Resonances red-shift towards the symmetry-protected BIC state as the pump power is reduced.

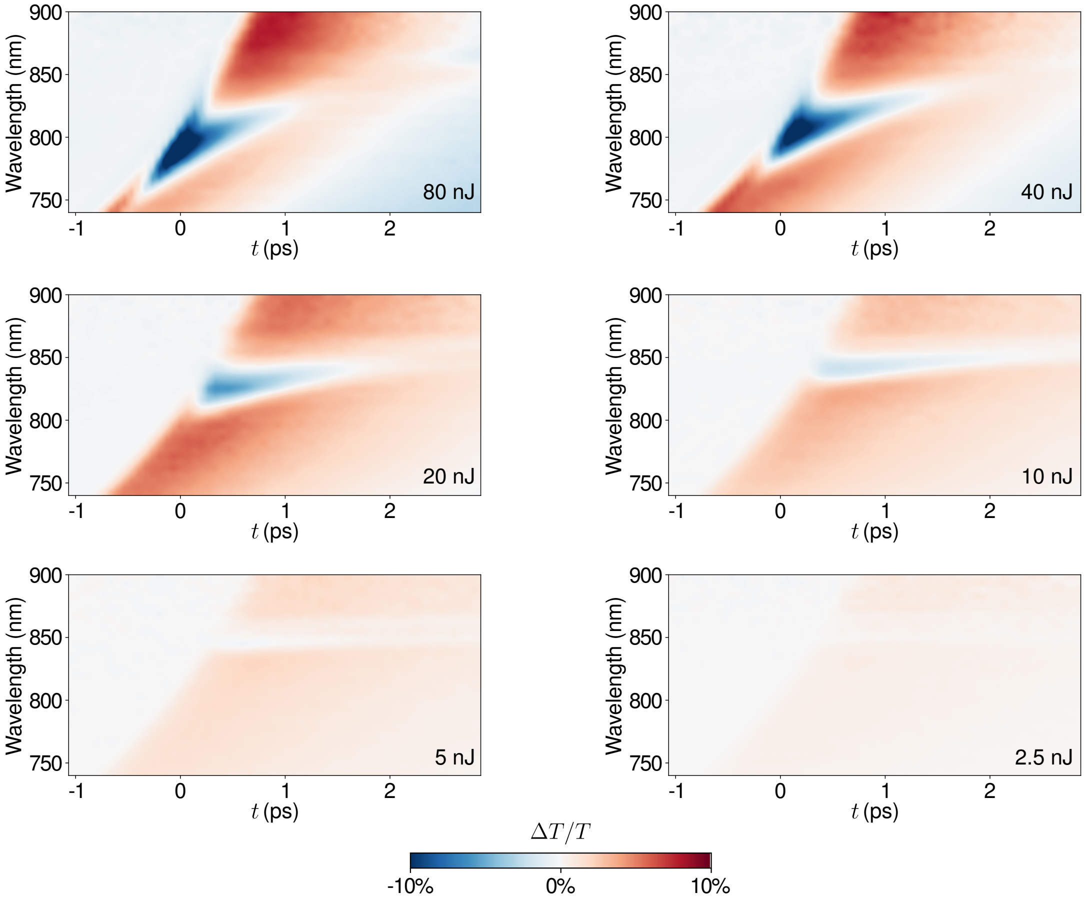

FIG. S9. Temporal evolution of ε-qBICs as a function of the pump pulse energy per arm (insets), $\lambda_{pump} = 410$ nm. Similar to the $\lambda_{pump} = 420$ nm case, resonances red-shift towards the symmetry-protected BIC state and decrease in amplitude modulation as the pump power is reduced.

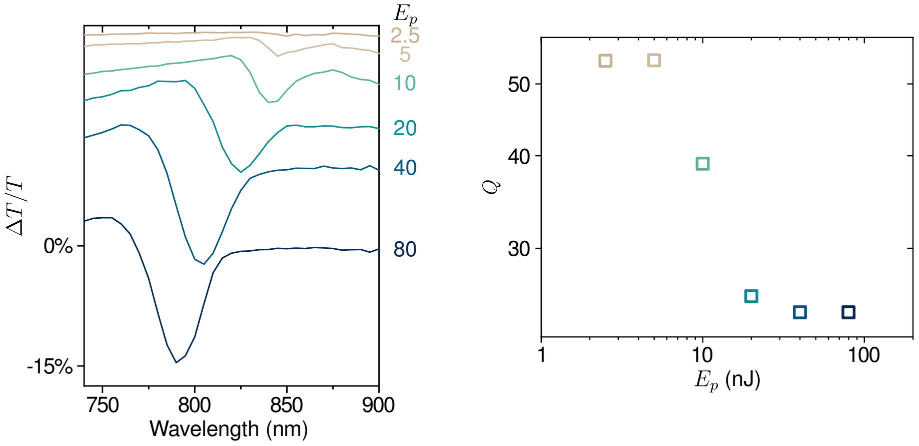

FIG. S10. Spectra (left) and respective Q-factors (right) of ε-qBICs as a function of the pump pulse energy per arm (E_p in nJ, indicated on the right hand side of the corresponding spectrum), λ_{pump} = 410 nm. Resonances blue-shift away from the symmetry-protected BIC state and increase in transmittance modulation for larger pump powers, being 80 nJ per pump arm the upper power limit of our setup for this wavelength.

Q VERSUS THE ASYMMETRY PARAMETER FOR A LOSSLESS METASURFACE

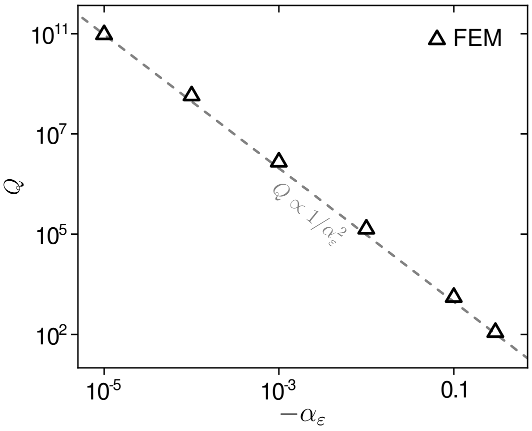

FIG. S11. Finite element method (FEM)-calculated Q of a lossless metasurface (triangles) vs the negative (due to a decreasing permittivity with increasing free-carrier concentration) of the asymmetry parameter α_ε, in agreement with the theoretical prediction (dashed grey line, $Q \propto 1/\alpha_\varepsilon^2$).

FITTING OF DIFFERENTIAL TRANSMITTANCE DATA

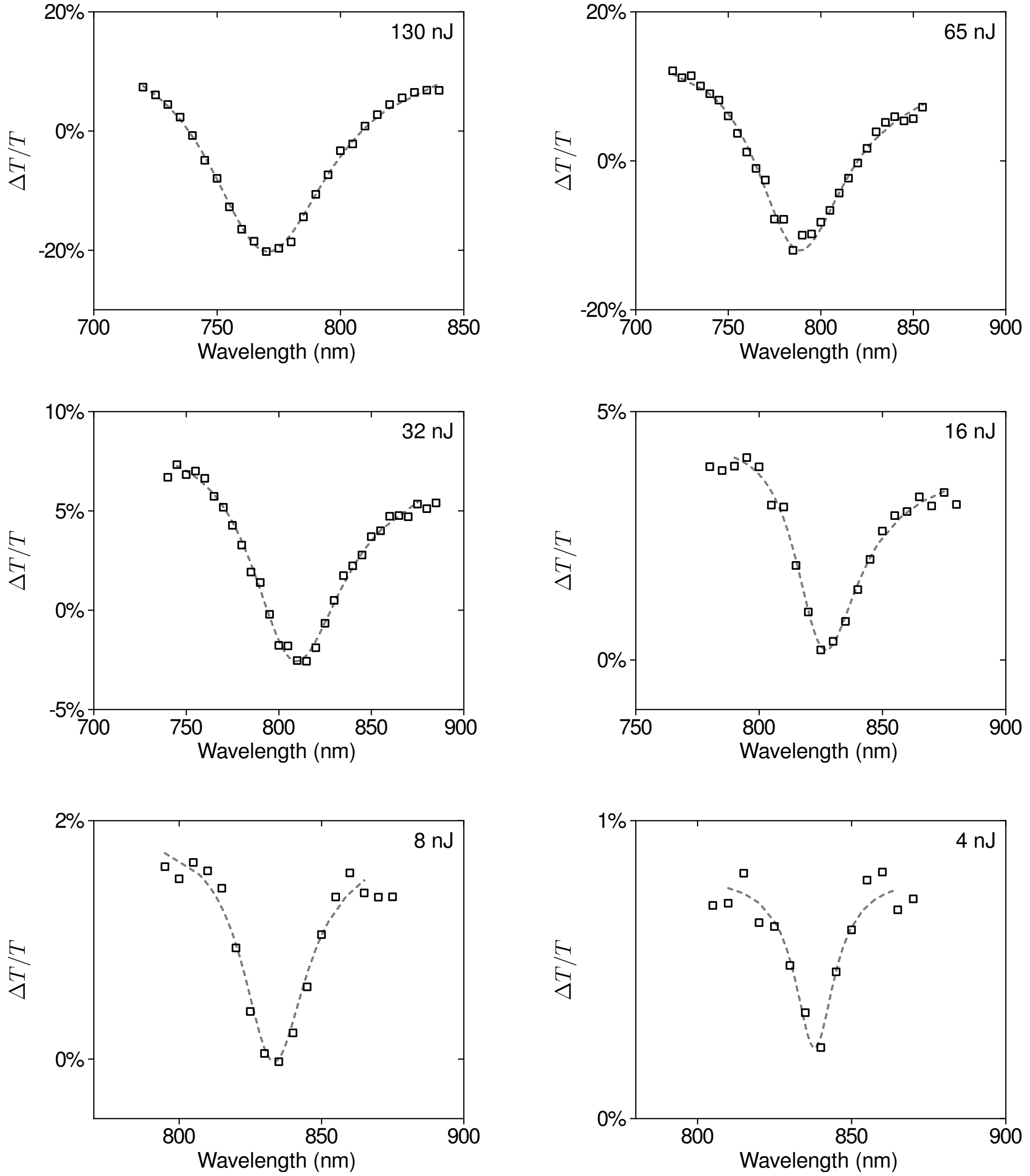

FIG. S12. Fitting of differential transmittance for different pump pulse energies (insets), $\lambda_{pump} = 420$ nm. A Breigt-Wigner-Fano (BWF) function $\frac{\Delta T}{T} = T_0 + \frac{H\left(1+\frac{\lambda-\lambda_c}{qw}\right)^2}{1+\left(\frac{\lambda-\lambda_c}{w}\right)^2}$ was used to fit (dashed lines) the data (open squares), where H is the height, q is the Fano asymmetry parameter, λ_c is the central wavelength of the resonance and w its width. The resulting Q-factor is calculated as $Q = \lambda_c/2w$.

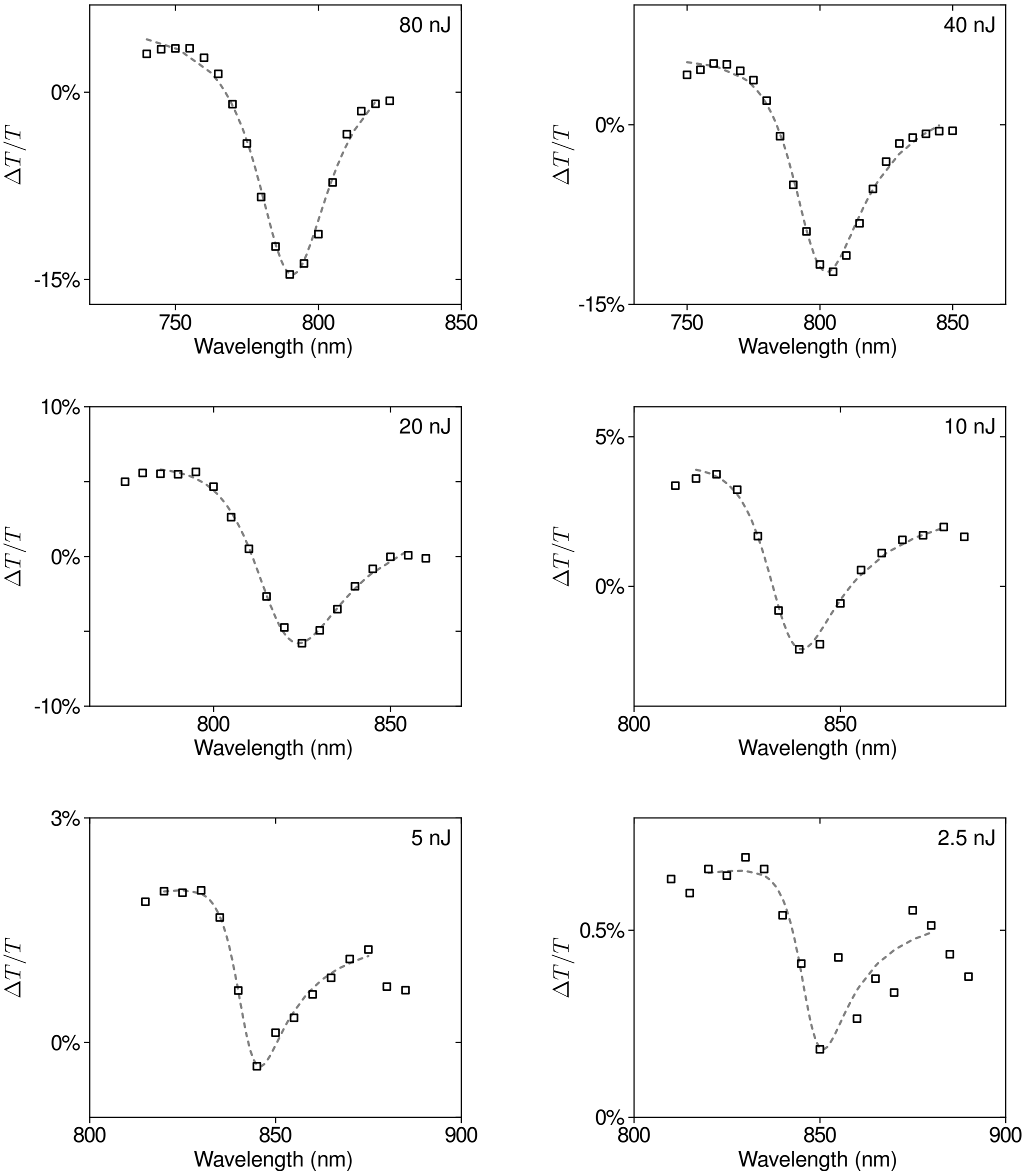

FIG. S13. Fitting of differential transmittance for different pump pulse energies (insets), $\lambda_{pump} = 410$ nm. A Breigt-Wigner-Fano (BWF) function was used to fit (dashed lines) the data (open squares).

PERMITTIVITY DATA OF SILICON FILMS

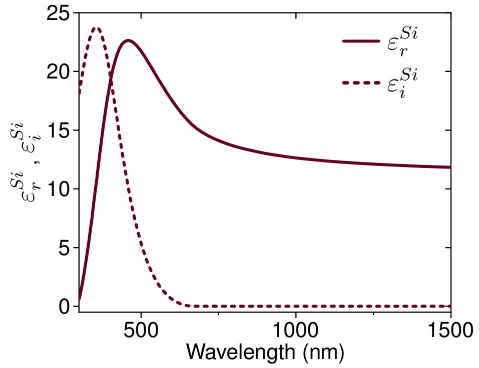

FIG. S14. Real (ε_r^{Si}, solid line) and imaginary (ε_i^{Si}, dashed line) parts of the permittivity of the fabricated a-Si films as a function of the wavelength, measured via ellipsometry.

PERMITTIVITY AND PERMITTIVITY ASYMMETRY AS A FUNCTION OF THE PUMP PULSE ENERGY

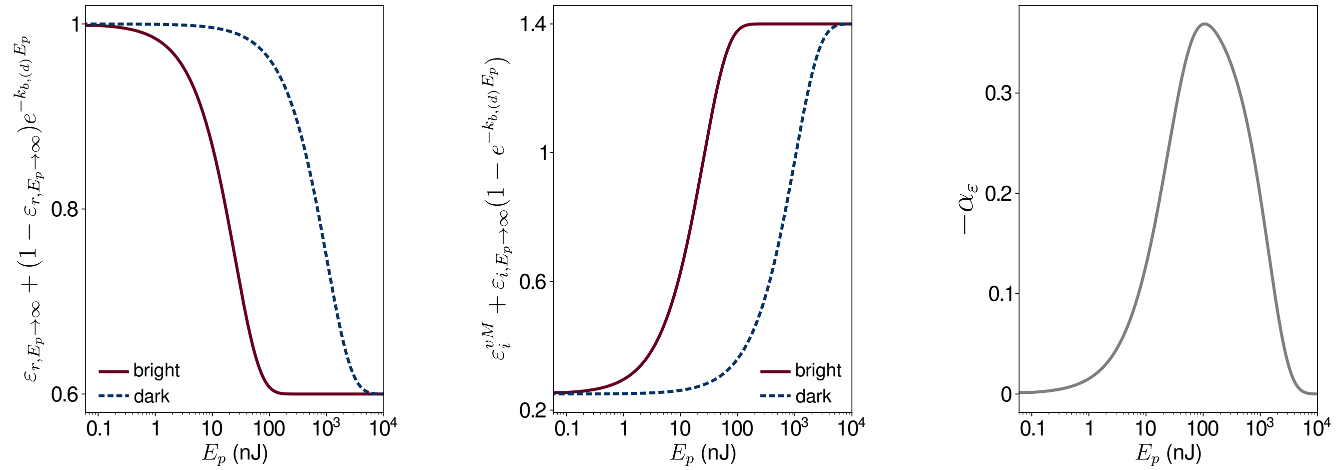

FIG. S15. Real (multiplicative, left) and imaginary (additive, center) permittivity terms of bright and dark fringes of the metasurface as a function of the pump pulse energy per arm (E_p). (right) $-\alpha_\varepsilon$ versus E_p. Values were obtained from the model shown in Eqn. 2 of the main manuscript. The parameters values used in model are $\varepsilon_{r,P\to\infty} = 0.6$, $\varepsilon_i^M = 0.25$, $\varepsilon_{i,P\to\infty} = 1.15$, $k_b = 0.04$ nJ^{-1} and $k_d = 0.001$ nJ^{-1}. $-\alpha_\varepsilon$ decreases for large pulse energies due to an absorption saturation of the bright fringes.

SPECTRUM VERSUS PUMP PULSE ENERGY

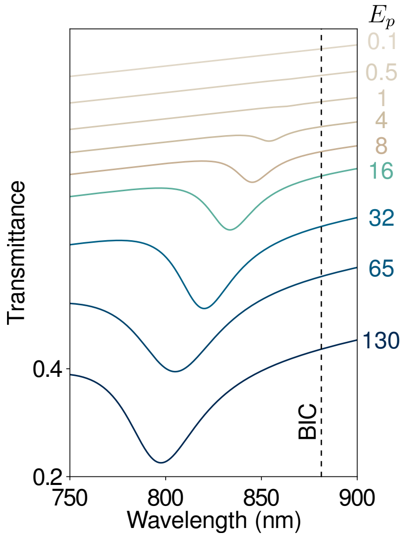

FIG. S16. Finite-difference time-domain (FDTD) method-calculated transmittance spectra as a function of the pump pulse energy (E_p) per arm (shown on the right). Permittivity parameters used in calculations are the same as stated in the caption of Fig. 2 of the main manuscript.

GMR AND ε-QBIC AT SMALL OPTICAL LOSSES

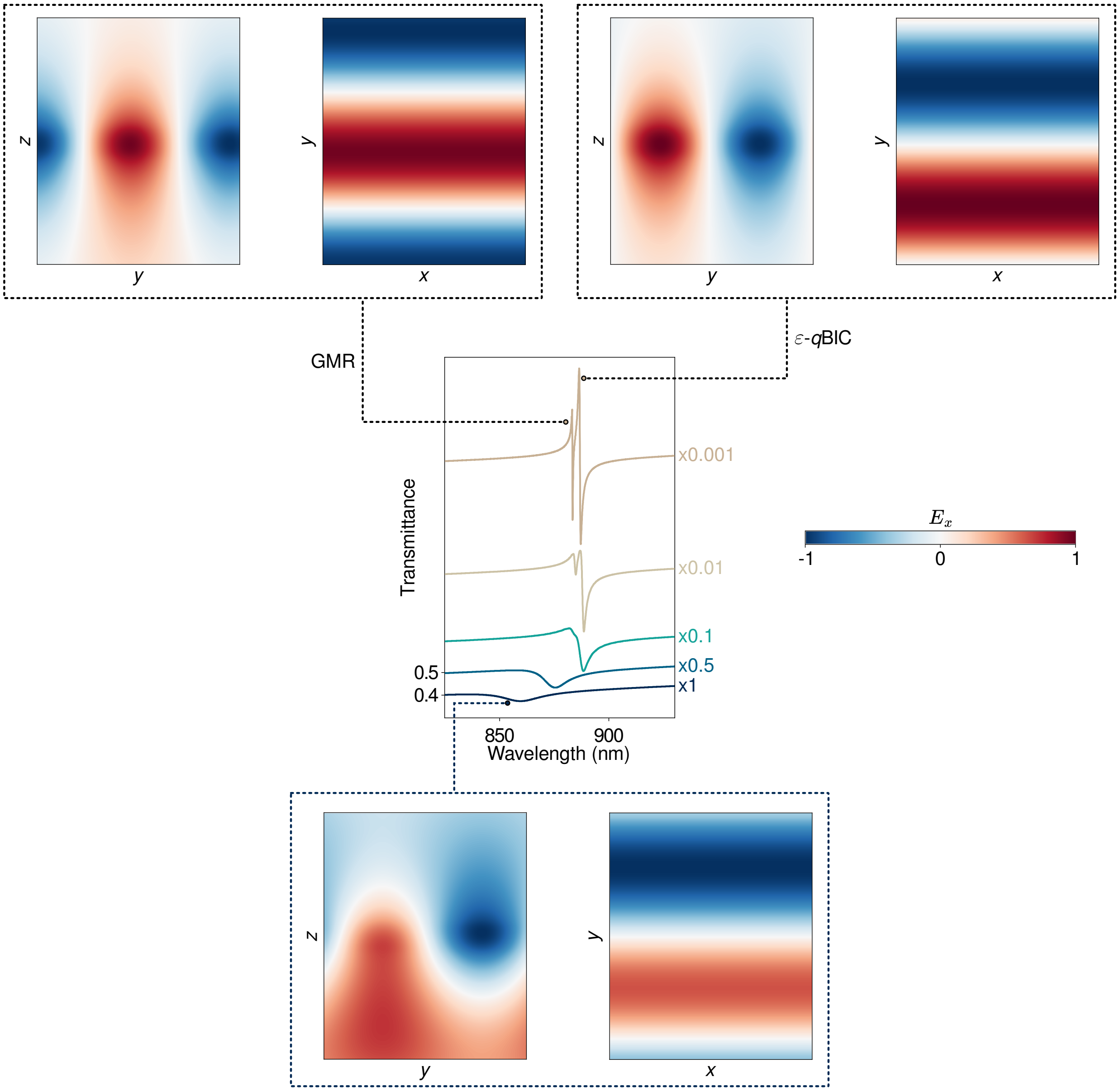

FIG. S17. FDTD method-calculated normalized near-field (E_x) and transmittance spectra as a function of imaginary permittivity of the fringes. Permittivity parameters used in calculations are the same as stated in the caption of Fig. 2 of the main manuscript, $E_p = 4$ nJ. However, the imaginary part of the permittivity (see Eq. 2 of the main manuscript) is multiplied by an arbitrary factor (shown on the right of the central graph). The reduction of optical losses shifts the resonance and turns it into two distinct and spatially-orthogonal optical modes (the GMR - top left, and the ε-qBIC - top right). The spectral signature of the GMR disappears as the optical losses are progressively increased, while the ε-qBIC is more resilient to losses, allowing its easier experimental observation. E_x at $\times 1$ losses (bottom panel) is characteristic of the ε-qBIC.

OPTICAL RESPONSE VERSUS THE POLARIZATION OF ONE OF THE PUMP BEAMS

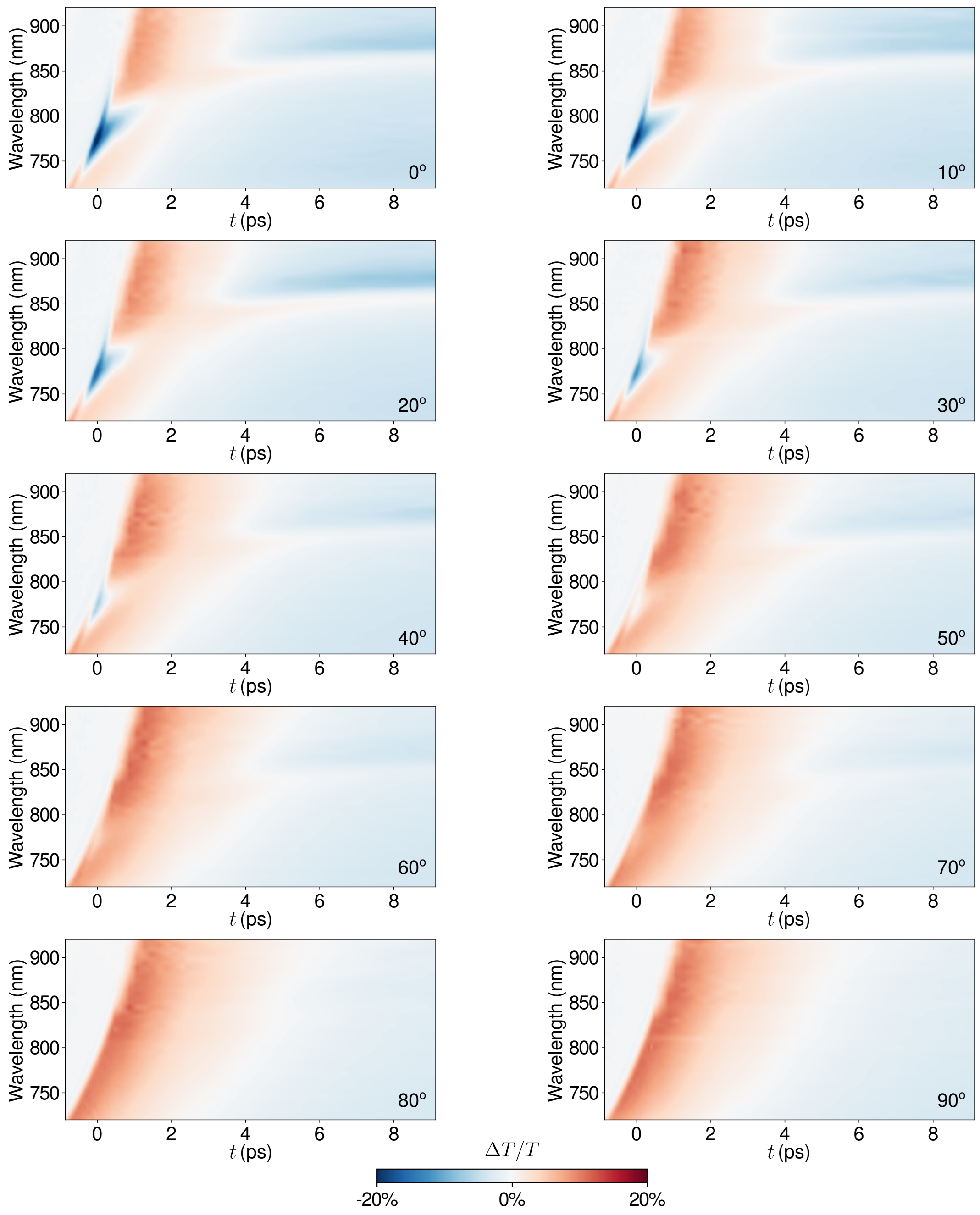

FIG. S18. Effect on the ε-qBICs of the relative polarization (shown in the insets) between the pump beams, controlled via a half-wave plate positioned in one of the pump arms (as shown in Fig. 1c of the main manuscript). As the relative polarization is increased towards an orthogonal configuration, the optically-induced metasurface generation is hindered, and the mode excitation is less effective. Measurements performed at $\lambda_{pump} = 420$ nm and 130 nJ pulse energy per arm.

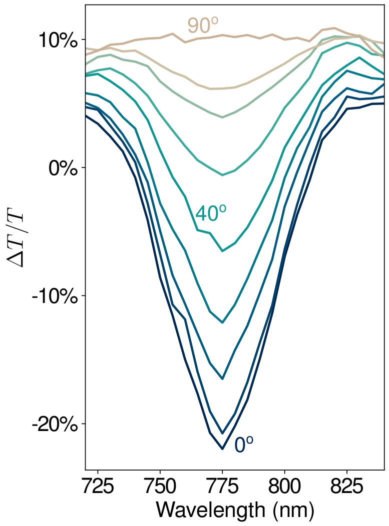

FIG. S19. Temporal cross-sections at $t = 0$ ps of modulated transmittance as a function of the relative polarization between the pump beams (selected angles shown along curves as inset).

TEMPORAL EVOLUTION OF ε-QBICS AS A FUNCTION OF THE PROBE POLARIZATION

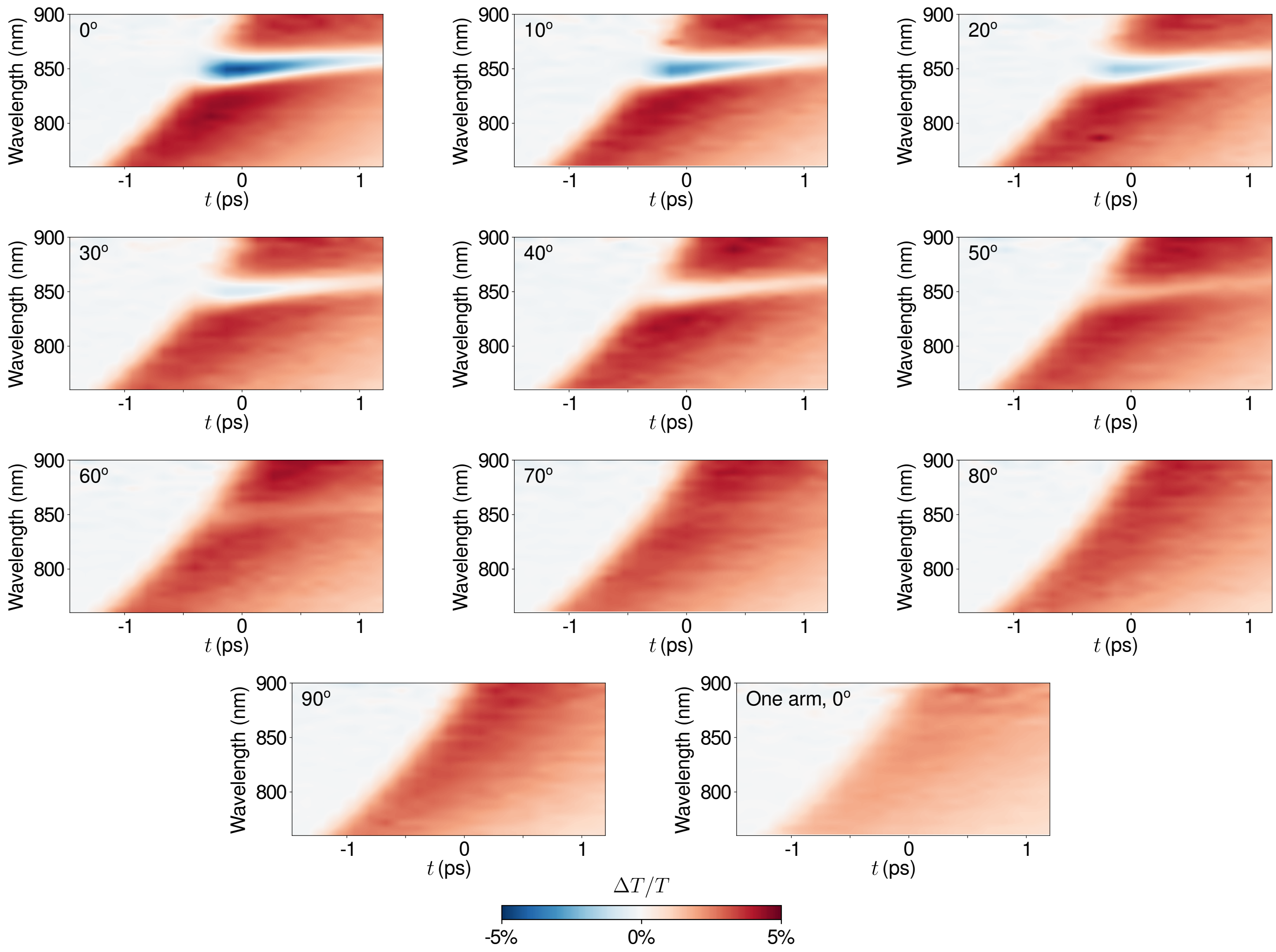

FIG. S20. Temporal evolution of ε-qBICs as a function of the probe polarization (insets) relative to the x-axis of the metasurface (along the ridges). Coupling to the ε-qBIC reduces as the relative polarization angle increases. When only one of the pump arms illuminates the thin film (a condition in which no metasurface is formed) no resonance is observed, even at a 0° relative polarization. Measurements performed at pump wavelength λ_{pump} = 420 nm and 16 nJ pulse energy per arm.

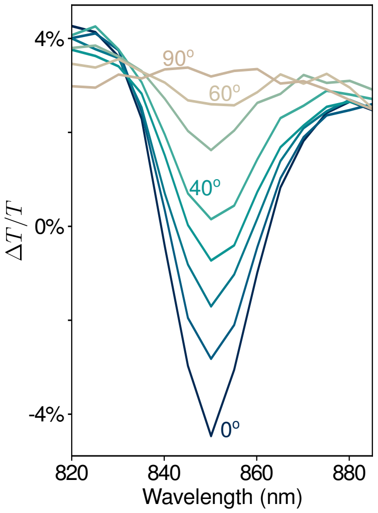

FIG. S21. Temporal cross-sections at $t = 0$ ps of differential transmittance versus probe polarization (inset) relative to the x-axis of the metasurface (along the ridges, see Fig. 1 of the main manuscript). Coupling to the ε-qBIC reduces as the relative polarization angle increases.

THIRD-HARMONIC GENERATION FOR DIFFERENT PUMP BEAMS CONFIGURATIONS

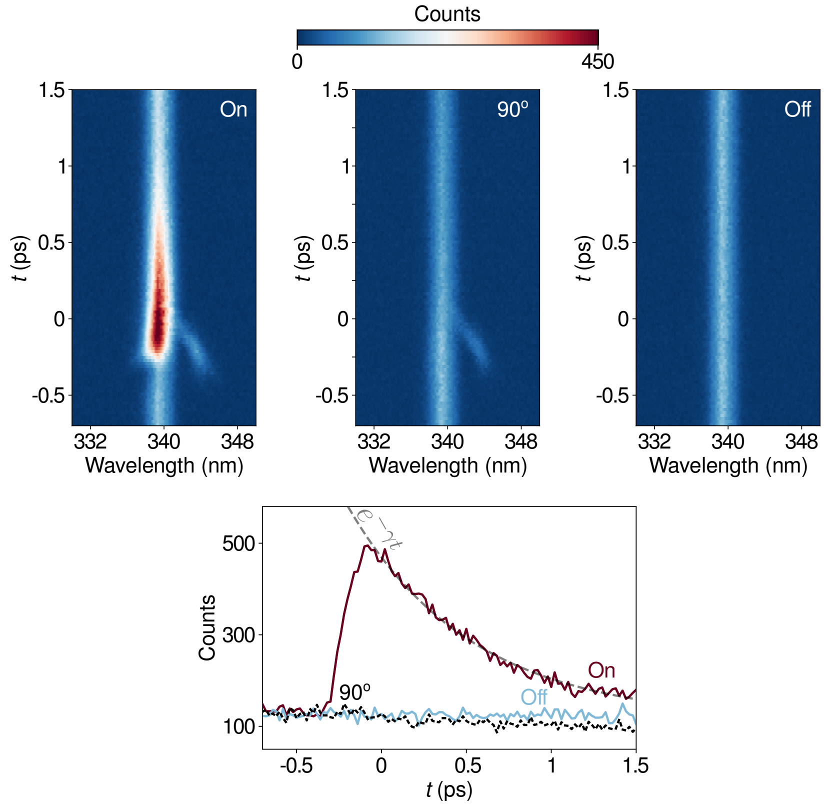

FIG. S22. (top) Third harmonic generation (THG) signal versus delay line position (in ps) for different pump configurations (insets). $\lambda_{fundamental} = 1020$ nm. THG enhancement is observed following the formation of the metasurface (0°, left) and the excitation of an ε-qBIC, while a nearly constant signal is obtained for cross-polarized (90°, center) and in the absence of the pump beams (Off, right). Spot at $\approx$ 343 nm in the left and central panels corresponds to the four-wave mixing signal (FWM, $\nu_{fwm} = \nu_{515nm} + \nu_{515nm} - \nu_{1020nm}$), a process due to the overlap of the ultrafast pump and probe beams. (bottom) Time trace of the THG signal at 339 nm for the corresponding configurations. THG enhancement decays exponentially (dashed grey line, $1/e \approx 0.72$ ps), along with the *quasi*-symmetry-protected resonance excited at low pump powers, not being a process limited by the Gaussian overlap of ultrafast beams. For cross-polarized pump beams (90°, center), a slight decrease in the THG signal is observed, attributed to increased losses in the film induced by the absorption of the pump beams. Pump pulse energy $E_{p,515nm} = 75$ nJ per arm, probe pulse energy $E_{p,1020nm} = 5$ nJ.

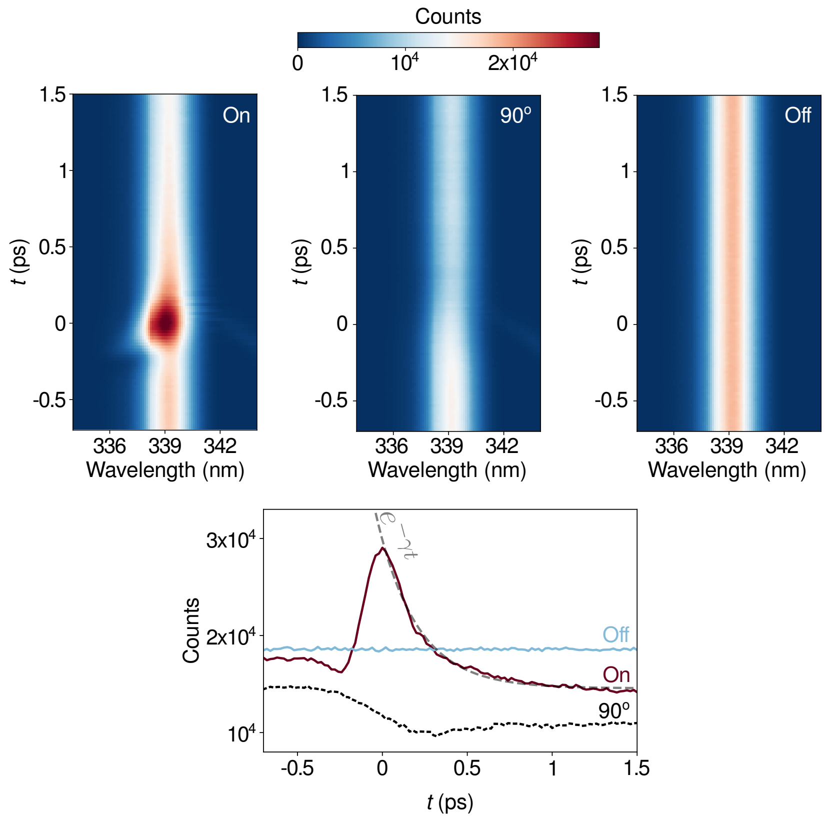

FIG. S23. Third harmonic generation (THG) signal at higher probe pulse energy versus delay line position (in ps) for different pump configurations (insets). $\lambda_{fundamental} = 1020$ nm. (bottom) Corresponding THG signal as a function of time at $\lambda_{THG} = 339$ nm. A similar trend observed at low powers is obtained at high powers, with a faster exponential decay of the THG enhancement process (grey dashed line, $1/e \approx 0.24$ ps). $E_{p,515nm} = 50$ nJ per arm, $E_{p,1020nm} = 9$ nJ.

THIRD-HARMONIC GENERATION VERSUS PROBE BEAM POLARIZATION

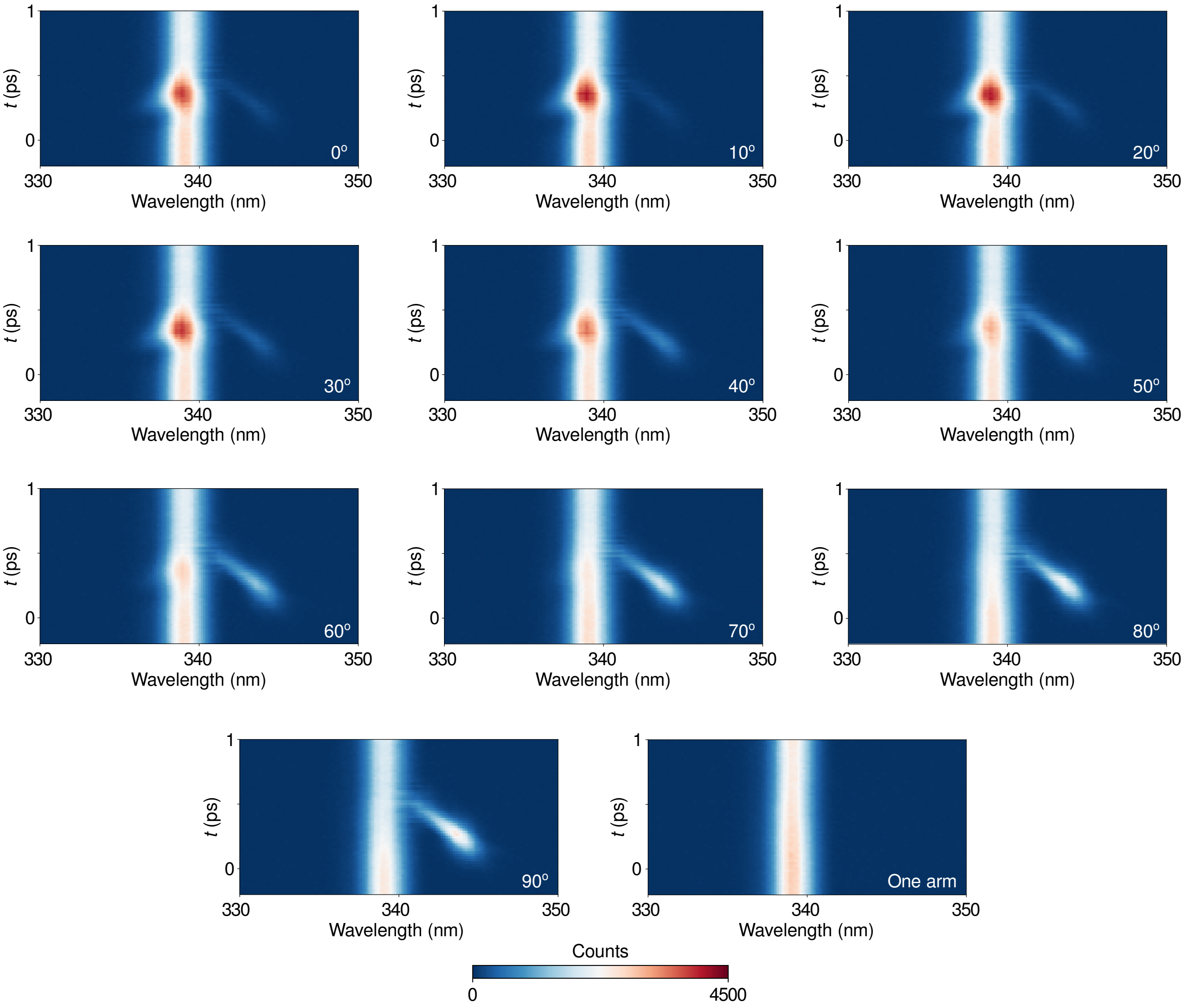

FIG. S24. THG and FWM signals vs delay line position (in ps) as a function of the relative polarization between the probe and the pump beams (inset). $\lambda_{fundamental} = 1020$ nm. The efficiency of THG (centered at ≈ 339 nm) decreases as the relative polarization angle between the probe and the pump beams increases. Red spot along the THG vertical strip corresponds to the excitation of an ε-qBIC and the enhancement of the THG signal. The efficiency of FWM ($\nu_{fwm} = \nu_{515nm} + \nu_{515nm} - \nu_{1020nm}$, at ≈ 343 nm), on the other hand, increases with increasing relative polarization. When only one pump arm is used, the efficiency of THG increases (relative to the 90° case, due to smaller losses), albeit with no enhancement, and no FWM signal is observed. $E_{p,515nm} = 50$ nJ per arm, $E_{p,1020nm} = 9$ nJ.

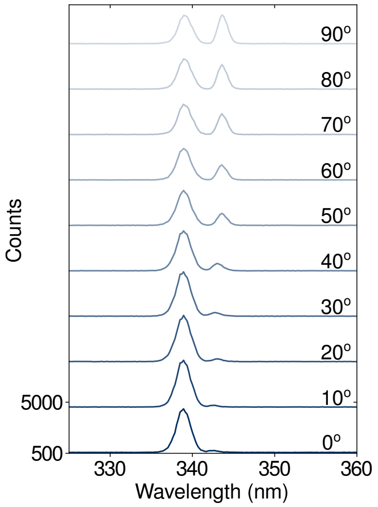

FIG. S25. TGH and FWM signals as a function of the relative polarization betweem pump and probe beams. A reduction of the THG signal (peak at ≈ 339 nm) occurs for larger relative polarizations while the red-shifted competing FWM process output increases. $E_{p,515nm} = 50$ nJ per arm, $E_{p,1020nm} = 9$ nJ.

THIRD-HARMONIC GENERATION VERSUS PROBE BEAM WAVELENGTH

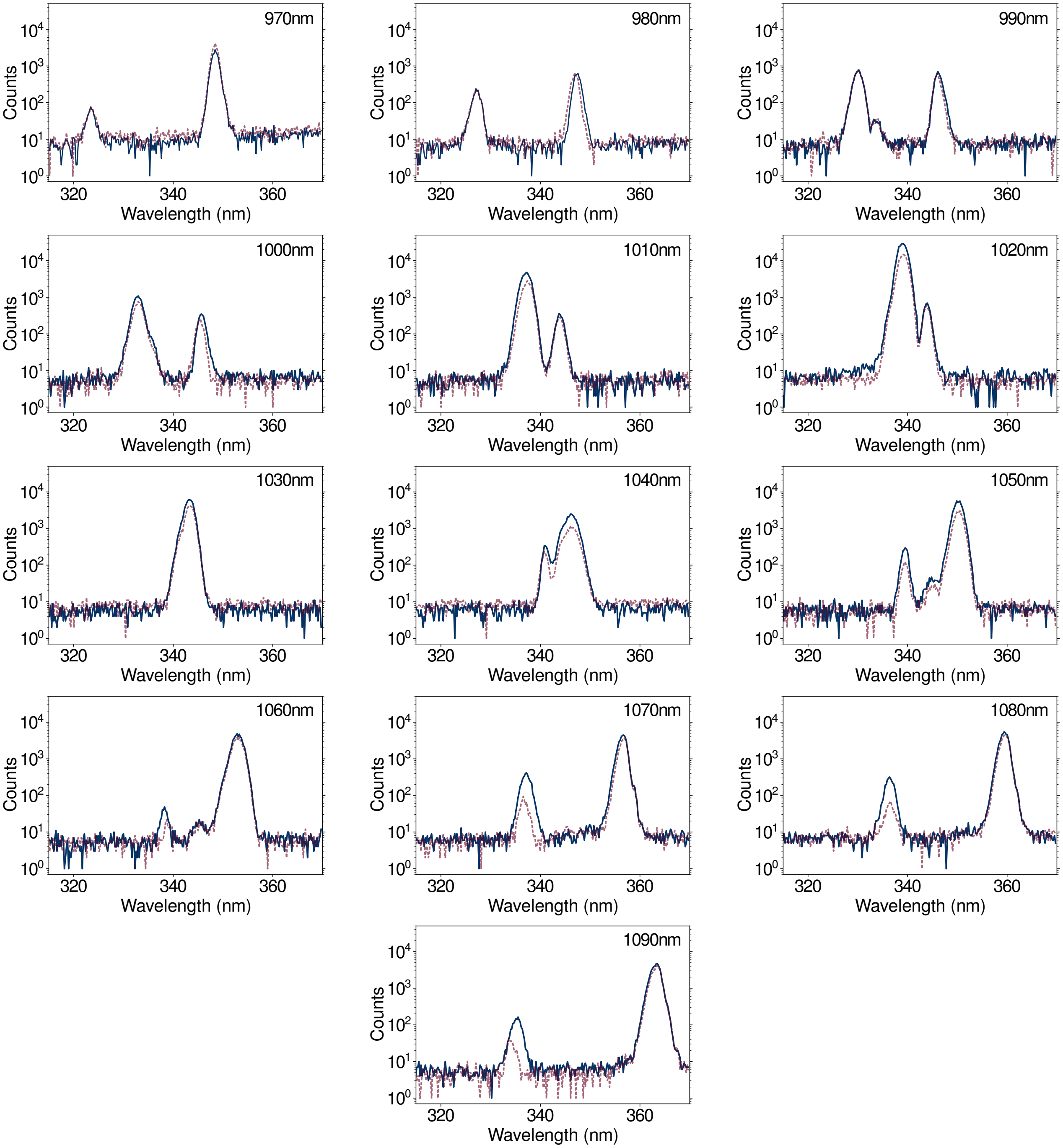

FIG. S26. THG and FWM signals vs probe wavelength (inset) for a 0° (blue) and a 90° (dashed red) relative polarization between pump beams ($\lambda_{pump} = 515$ nm). THG and FWM red- and blue-shift, respectively (crossing at $\lambda_{probe} = 1030$ nm), for longer probe wavelengths. Note the logarithmic scale in the y-axis (Counts).

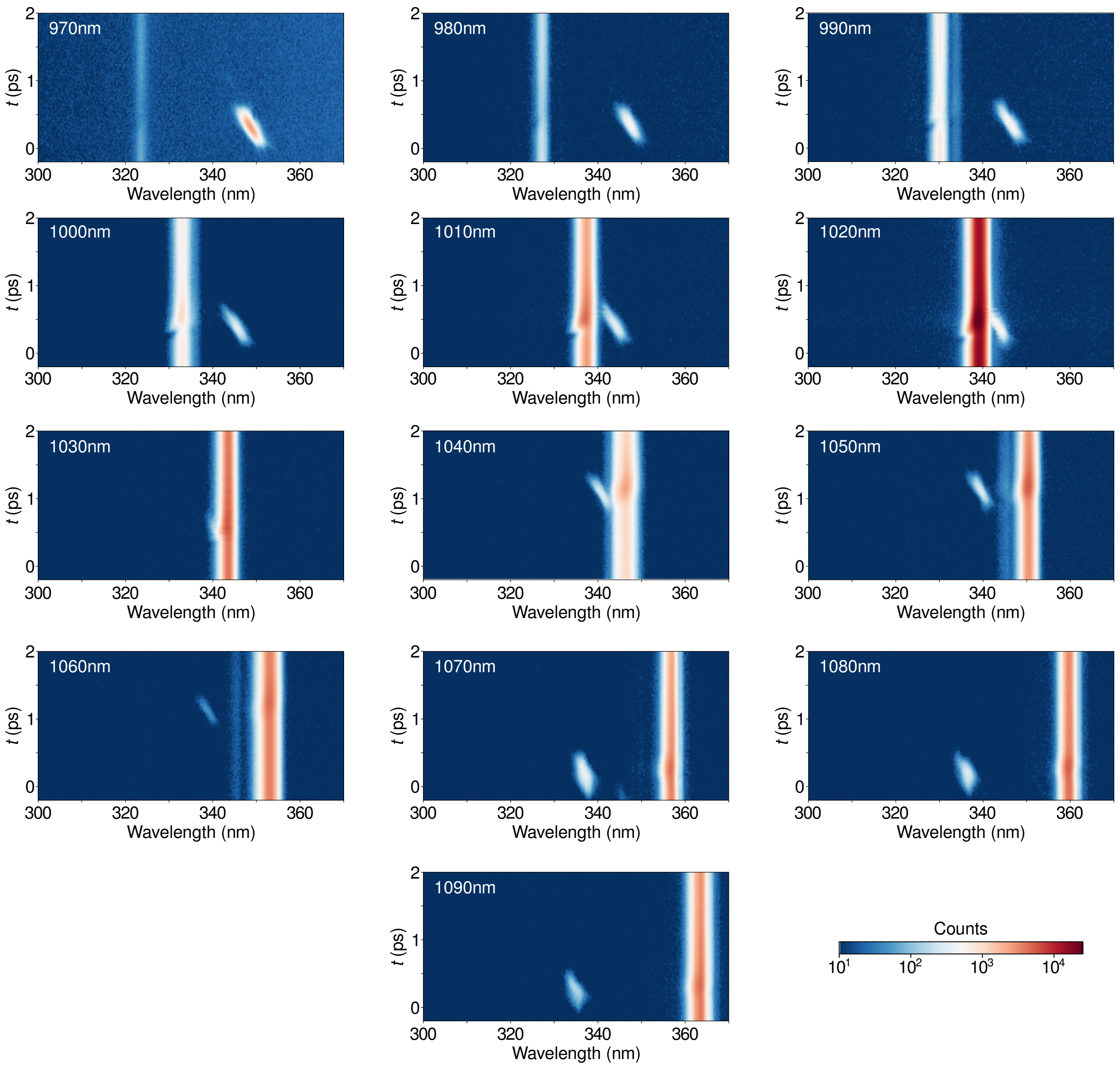

FIG. S27. THG and FWM signals vs delay line position (in ps) as a function of the probe wavelength (inset). Here a 0° relative polarization between the pump beams is used (thus forming the metasurface). Note the logarithmic scale in the colormap (counts). $E_{p,515nm} = 50$ nJ per arm, $E_{p,probe} = 9$ nJ. The simultaneous arrival of beams (red spot along the THG vertical strip) changes in time for $\lambda_{probe} = 1040$ nm and $\lambda_{probe} = 1070$ nm as the OPA changes its output beam between signal and idler. The fainter vertical stripes corresponds to both signal and idler beams being present, leading to two closely-spaced THG signals.

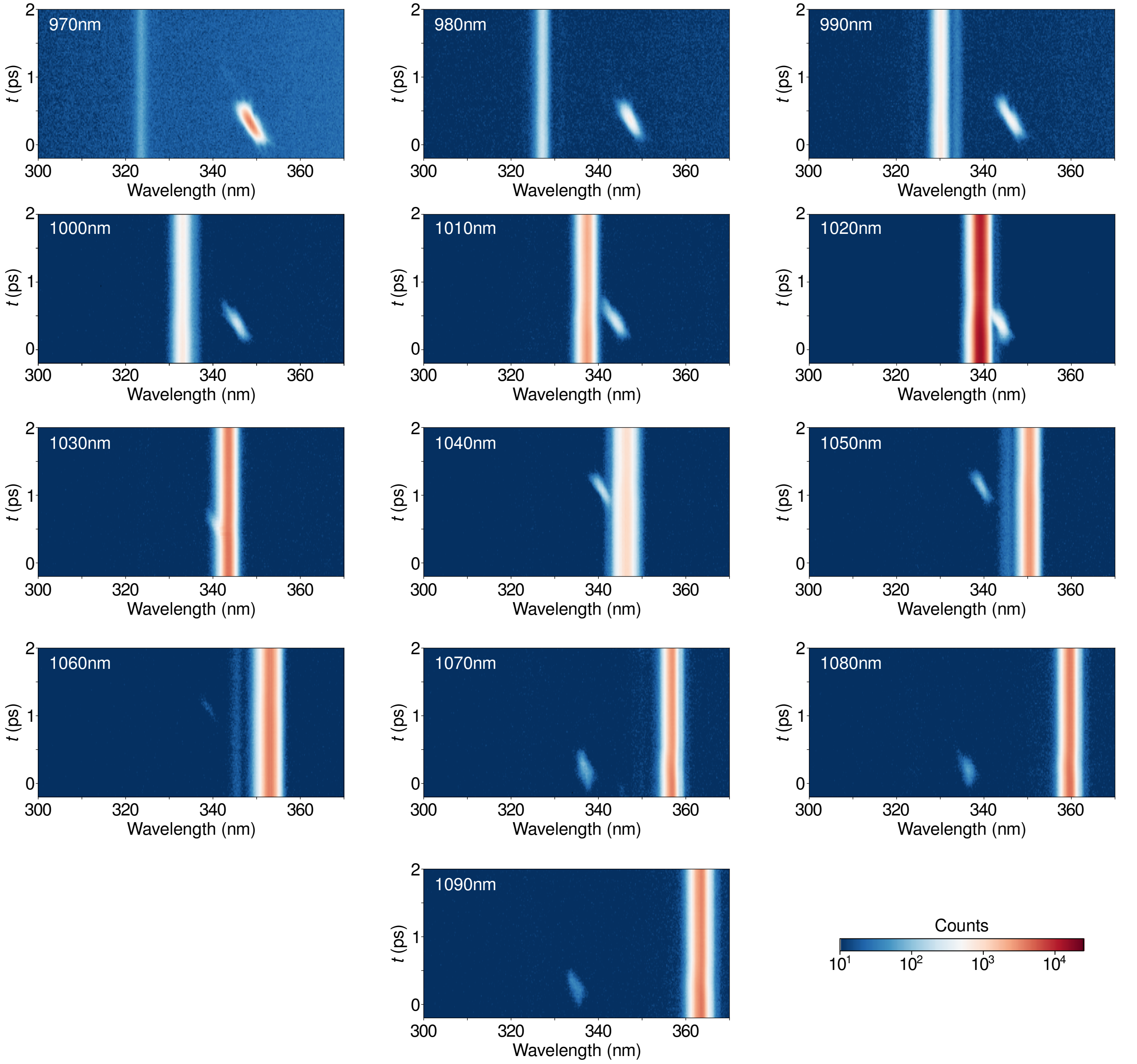

FIG. S28. THG and FWM signals vs delay line position (in ps) as a function of the probe wavelength (inset). Here a 90° relative polarization between the pump beams is used (absence of the metasurface), reducing the harmonic generation efficiency. Note the logarithmic scale in the colormap (Counts). $E_{p,515nm} = 50$ nJ per arm, $E_{p,probe} = 9$ nJ.

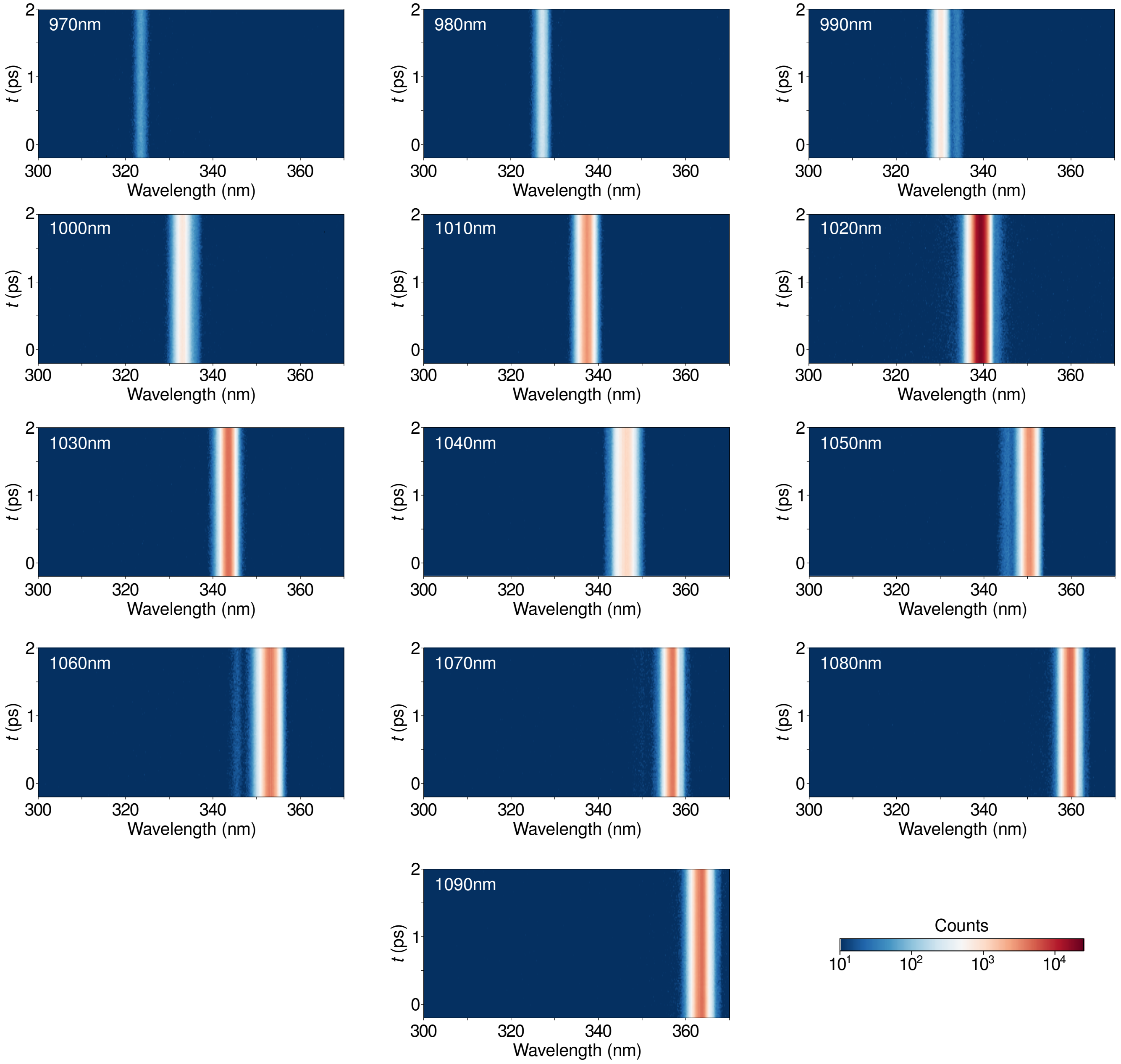

FIG. S29. THG and FWM signals vs delay line position (in ps) as a function of the probe wavelength (inset) in the absence of the pump beams. As the 515 nm photons are not present, the FWM signal vanishes. Note the logarithmic scale in the colormap (Counts). $E_{p,probe} = 9$ nJ.
